# Improved ascertainment of modes of HIV transmission in Ukraine indicates importance of drug injecting and homosexual risk

**DOI:** 10.1186/s12889-020-09373-2

**Published:** 2020-08-26

**Authors:** Kostyantyn Dumchev, Marina Kornilova, Roksolana Kulchynska, Marianna Azarskova, Charles Vitek

**Affiliations:** 1 Ukrainian Institute on Public Health Policy, Kyiv, Ukraine; 2 Alliance for Public Health, Kyiv, Ukraine; 3 Division of Global HIV and TB, Center for Global Health, U.S. Centers for Disease Control and Prevention, Kyiv, Ukraine; 4 Division of Global HIV and TB, Center for Global Health, U.S. Centers for Disease Control and Prevention, Atlanta, GA USA

**Keywords:** HIV epidemiology, HIV transmission, HIV risk factors, Injecting drug use, Ukraine

## Abstract

**Background:**

It is important to understand how HIV infection is transmitted in the population in order to guide prevention activities and properly allocate limited resources. In Ukraine and other countries where injecting drug use and homosexuality are stigmatized, the information about mode of transmission in case registration systems is often biased.

**Methods:**

We conducted a cross-sectional survey in a random sample of patients registered at HIV clinics in seven regions of Ukraine in 2013–2015. The survey assessed behavioral risk factors and serological markers of viral hepatitis B and C. We analyzed the discrepancies between the registered mode of transmission and the survey data, and evaluated trends over 3 years.

**Results:**

Of 2285 participants, 1032 (45.2%) were females. The proportion of new HIV cases likely caused by injecting drug use based on the survey data was 59.7% compared to 33.2% in official reporting, and proportion of cases likely acquired through homosexual transmission was 3.8% compared to 2.8%. We found a significant decrease from 63.2 to 57.5% in the proportion of injecting drug use-related cases and a steep increase from 2.5 to 5.2% in homosexual transmission over 3 years.

**Conclusions:**

The study confirmed the significant degree of misclassification of HIV mode of transmission among registered cases. The role of injecting drug use in HIV transmission is gradually decreasing, but remains high. The proportion of cases related to homosexual transmission is relatively modest, but is rapidly increasing, especially in younger men. Improvements in ascertaining the risk factor information are essential to monitor the epidemic and to guide programmatic response.

## Background

The “Know your HIV epidemic” approach introduced and guided by the Joint United Nations Programme on HIV/AIDS has become a critical part of the HIV/AIDS response [1]. Understanding trends in HIV transmission in high-risk key populations is crucial for optimal allocation of limited prevention resources.

Available data about modes of HIV transmission are often suboptimal not only because of the stigma associated with HIV risk behaviors but also because of systemic flaws in reporting systems. Most case-reporting systems collect information on modes of HIV transmission based on patients’ self-reporting, which is usually neither accurate nor complete because this requires disclosure of sensitive and potentially stigmatizing information [2, 3]. For example, in the US, completion of risk factor information in case report forms submitted to the HIV/AIDS Reporting System is not mandatory, which contributed to < 80% of cases among men being reported within a known transmission category in 2014 [4]. Despite the relatively low level of stigma in the US, men who have sex with men (MSM) and injecting drug use behaviors were underreported: about 77% of 6891 men who did not report a transmission category were estimated to be MSM and 8% to be persons who inject drugs (PWID) [4].

In Ukraine, which has the second largest HIV epidemic in Europe [5], the case registration system captures nearly all cases with confirmed HIV infection, but introduces two potential biases in assessing the mode of transmission. First, the registration form, filled once at the time of diagnosis confirmation, does not include fields for specific risk factors, and does not provide guidelines for structured collection of risk factor data. The substantial stigma toward injecting drug use and MSM [6] may lead to underreporting of these behaviors by the patients and incorrect assumptions by healthcare providers. Second, unlike in the US, where CDC developed a hierarchy of transmission categories [7], Ukraine’s system lacks guidance on how to rank multiple risk factors and establish the probable mode of transmission. As a result, the indirect evidence indicates that modes of transmission are substantially misclassified [8]. An analysis of hepatitis C virus (HCV) prevalence among HIV-positive men in 2009 suggested that as many as 34.5% of men registered with heterosexual exposure as the mode of transmission could in fact have acquired HIV through injecting drug use [8]. Extrapolation of national biobehavioral survey data suggests that another 8.3% of these men could be MSM [8, 9]. A triangulation study, combining all existing data sources, suggested that HIV transmission remained linked to PWID and their sexual partners [10].

According to official reported case registration data, heterosexual exposure was the dominant mode of HIV transmission in Ukraine in 2008, accounting for 70.1% of 12,893 adults (aged ≥15 years) registered in 2015 [11]. Injecting drug use, once the main driver of the HIV epidemic, has decreased to 26.8%. The number of cases officially attributed to homosexual exposure increased steadily, from 20 in 2005 to 368 in 2015. These trends and the fact that the absolute number and rate of new HIV infections started decreasing in 2012, following the overall trend in Eastern Europe (excluding Russia) [5], may suggest that injecting drug use is no longer, and that transmission among MSM is not yet, a major factor in the Ukrainian HIV epidemic. Additionally, the high proportion of cases attributed to heterosexual exposure and declining levels of HIV among female sex workers [12] would support directing resources toward prevention of heterosexual transmission in the general population. However, if the magnitude of misclassification of the mode of transmission is as significant as previous evidence suggests, these conclusions would be invalid.

The primary goal of this study was to assess the risk factors among patients diagnosed with HIV using a sensitive algorithm and estimate the degree of misclassification in the registered mode of HIV transmission. This evidence will inform improvement in the official HIV case registration system including the update of the case reporting form and training of healthcare providers involved in assessing modes of transmission. The secondary goal of the study was to assess the changes in the main transmission categories from 2013 to 2015 and determine the epidemic trends.

## Methods

### Study design and population

In Ukraine, individuals who screen positive for HIV at any community- or facility-based testing sites are referred to a network of government HIV clinics for confirmatory testing, diagnosis and registration. To assess the HIV risk factors and biological markers of transmission, we conducted a cross-sectional survey among adult patients who were officially registered at all clinical facilities in three most recent years before the study, from 2013 to 2015.

### Sampling

We used a two-step random sampling approach to recruit participants into the study. At the first step, we selected seven out of 27 administrative regions of Ukraine using a random number generator. To estimate the prevalence of HIV risk factors with a 95% confidence level, 5% margin of error and assuming a survey design effect of 2.0, the target sample size was 775 per each year. To ensure representation of regions with different sizes of the epidemic (which could potentially be associated with case registration practices), we sorted the list of regions by the number of HIV cases reported in 2013 [13] and chose each fourth unit starting from a randomly generated number. The target sample size was divided across the seven regions proportionally to the number of cases reported in 2013 (Supplement Table S1).

Since there was no significant change in the number of registered patients in the selected regions over time, we chose to sample patients from three equal 3-month periods (October–December) to investigate the change in mode of transmission over time.

At the second step, we used a random sampling approach to recruit survey participants. We extracted data from patient registration forms to create a de-identified registry of patients diagnosed during the specified three periods (Supplement Table S2). The data included personal within-clinic ID code, sex, year of birth, date of registration, date of discharge (if applicable) and reason of discharge, dates of HIV tests used for diagnosis, registered mode of transmission, and stage of HIV disease at the time of diagnosis. In a random order, patients from the registry were contacted and invited to participate in the survey. All patients who attended the study appointment provided written informed consent.

### Data collection

After providing informed consent, patients completed an anonymous survey to determine the patients’ HIV risk factors 10 years before finding out about their HIV-positive status. The survey instrument was developed by the investigators specifically for this study, and was pilot tested on 10 patients to adjust wording that could be misinterpreted. The questionnaire (see Supplementary File 1) included the following sections: sociodemographic information, sexual risk factors and protective behaviors, injecting drug use history, and history of sexually transmitted or bloodborne infections. The survey was administered by trained interviewers using the REDCap electronic data capture tools hosted at Ukrainian Institute on Public Health Policy [14]. To limit self-reporting bias, interviewers were trained in psychological techniques to enhance rapport with respondents. Although the interviews took place in the HIV clinics, the interviewers were not clinic staff, and the information collected was not disclosed to any clinic staff. No one from the local research teams except the interviewers had access to the REDCap database. The survey data did not include any personal identifiers and were linked to the patient recruitment logs and registries using a participant ID. Before the interview, the interviewers explained that they did not have access to personal information in the clinical records and that the clinical staff did not have access to the interview data. The interview on average took 15 min to complete.

After the interview, participants provided a blood sample for hepatitis B virus (HBV) and HCV testing. Samples were centrifuged, and plasma samples were frozen. After recruitment, the frozen samples were shipped to a national viral hepatitis reference lab at the Kyiv City AIDS center. The samples were tested for biomarkers of HBV and HCV infection using the ARCHITECT-i1000SR Immunoassay Analyzer and the following reagent kits: ARCHITECT HBsAg, ARCHITECT Anti-HBs, ARCHITECT Anti-HBc, ARCHITECT Anti-HBc IgM and ARCHITECT anti-HCV. Results were reported both to the study investigators and to regional HIV clinics to inform clinicians and the patients about the results of the tests.

Data were collected between March and October 2016.

### Data analysis

The descriptive analysis focused on the distribution of the registered mode of transmission in the official reports and verified registry and on the distribution of HIV risk factors among the survey participants. The data were disaggregated by sex, year of registration, and region. HIV surveillance data represents a census of HIV diagnoses [15] for the selected regions; therefore, no confidence intervals are presented.

In clinical records and official reports, registered modes of transmission were heterosexual exposure, homosexual exposure, injecting drug use, blood product transfusion, transplantation of organs or tissues, other medical exposure, occupational exposure, other non-medical exposure, confirmed mother-to-child, unconfirmed mother-to-child, and unknown. Mother-to-child transmission cases were excluded from this study. We combined all categories with few cases (except heterosexual exposure, homosexual exposure, and injecting drug use) into an “other” category.

For each risk factor identified in the survey, we created a dichotomous variable based on one or more questions. Some participants were not consistent in responding to different questions addressing the same risk factor; therefore, we constructed logical formulas defining absence or presence of the factor (Table 1). Both in descriptive analysis and in hypothesis testing, we treated these variables as not mutually exclusive, recognizing that one person may be exposed to more than one factor at the same time.

**Table 1:** Logical formulas for risk factor definitions

Heterosexual	heterosexual exposure (regardless of having homosexual exposure)^a^ OR having one or more partners of the opposite sex^a^ OR self-reporting being infected through heterosexual exposure
High-risk heterosexual	having heterosexual exposure (defined above) AND [having had a sexual partner who injects drugs^a^ OR having had heterosexual contact with an HIV-positive person^a^ OR giving or receiving money or drugs for sex^a^ OR [having had a sexual partner who was bisexual^a^ AND being female]]
Injecting drug use	injecting illicit drugs at least once^a^ OR self-reporting injecting drug use as the most likely mode of HIV transmission
Homosexual	being male AND [having sexual contact with men at the present time OR having one or more male sexual partners^a^ OR having a sexual partner of the same sex^a^ OR having homosexual contact with an HIV-positive person^a^ OR self-reporting homosexual exposure as the most likely mode of HIV transmission]
Nosocomial	having had blood or blood product transfusion^a^ OR having had organ or tissue transplantation^a^ OR having had in vitro fertilization^a^ OR self-reporting being infected through medical procedures
Skin penetration	reporting intentional skin penetration (tattoo, scarring, or other practices)^a^ OR having been exposed to another person’s blood through damaged skin or mucosa^a^ OR reporting being infected in an occupational or non-occupational accident with skin penetration
Sexually transmitted infections	self-report on having HBV OR gonorrhea OR syphilis OR genital herpes OR proctitis OR other STI at any time before finding out about HIV-positive status
Exposure to HCV	positive test for anti-HCV antibodies
Exposure to HBV	positive test for HBsAg OR positive test for AntiHBc antibody

^a^during 10 years before finding out about HIV-positive status

We constructed a summary variable representing the most probable mode of transmission based on the survey responses. Given the strong correlation between HCV and injecting drug use, presence of anti-HCV antibodies was considered a marker of injecting drug use-related transmission. If no anti-HCV antibodies were detected, the survey-based mode of transmission was based on self-reported behavior. If only one risk factor was reported by a participant, the survey-based mode of transmission was assigned the corresponding value. If a participant reported multiple exposures, the survey-based modes of transmission took the value of a risk factor associated with greater probability of transmission per act [16] and higher level of prevalence in respective key populations [12] in the following hierarchy: injecting drug use, homosexual exposure between men, heterosexual exposure, and other. We did not create more detailed or mixed categories, such as those developed by CDC and other authors [17], to enable comparison with Ukrainian registered modes of transmission data. Instead, we present prevalence of all possible two risk factor combinations.

Participants who refused to answer one or more questions required for determining the survey-based mode of transmission were excluded from the analysis of modes of transmission but were retained in the dataset for analysis of other variables.

#### Sensitivity analysis

To assess the randomness of the sampling approach and thus the representativeness of the survey sample, we compared the registered modes of transmission distribution in the survey sample and the rest of the patients in the registry who did not participate in the survey. The significance of difference for each registered mode of transmission was tested using Chi-square tests.

#### Hypotheses testing

The main research question was whether there is a difference between the proportion of patients in corresponding registered modes of transmission and survey-based modes of transmission categories. Since these two variables were measured using the same participants and could be considered related, we used the McNemar test to determine the significance of difference between proportions for each major mode of transmission (heterosexual exposure, injecting drug use, homosexual exposure, and other). To assess the extent of agreement between the registration data and survey-based determination of mode of transmission on individual level, we computed Cohen’s Kappa and corresponding *p*-value. We used the Mantel-Haenszel test for trend (for 2 × r tables) to test the significance of change in the proportion of main transmission categories and prevalence of risk factors over time [18].

To determine the accuracy of the official paper-based reporting system, we used the Chi-square test to determine difference in the distribution of the main modes of transmission between the reporting forms and the verified registry.

*P*-values less than 0.05 were considered statistically significant. Statistical analysis was done using SPSS for Windows version 23 (IBM Corporation, Armonk, NY USA).

#### Extrapolation

An adjusted distribution of modes of transmission among total national cases registered in 2015 was imputed by extrapolating the magnitude of misclassification observed in our sample. For each mode of transmission category, we calculated an extrapolation coefficient as a ratio of the proportion observed in the survey to the proportion in the registry. The coefficients were then applied to the reported number of cases within each category to calculate percentages representative of the adjusted modes of transmission among all HIV cases registered in 2015.

## Results

### Recruitment

A total of 3913 new HIV cases, excluding cases of mother-to-child transmission, were reported in the seven study regions during the three October–December periods in 2013, 2014, and 2015 (*N* = 1421, 1209, and 1283, respectively).

We verified and extracted data from 3627 patient registration forms into the study registry. In two regions, Lviv and Dnipropetrovsk, data from deceased or transferred patients were unavailable for extraction. In three regions, the study team found more registration forms (up to 20%) for patients registered within the study period than were included in the official registration reports.

Among the patients included in the registry, 9.2% were deceased, 1.5% had moved outside of the study region, 2.1% were incarcerated, and 9.3% were lost to follow-up. Of 2567 patients who were contacted, 2285 agreed to participate in the study, yielding an 89.0% response rate. Overall, we recruited 63.0% (50.0–88.0% across the regions, data presented in Supplement Table S2) of all patients in the verified registry.

The sample consisted of 54.8% men and 45.2% women. Median age at registration was 35 years (standard deviation [SD], 8.85). Of the study participants, 2260 answered definitively all questions required to construct the survey-based modes of transmission variable.

### Sensitivity results

Supplement Table S3 shows the distribution of registered modes of transmission in official reports and among patients in the registry who were and were not recruited. The recruited sample had a lower proportion of patients who were registered with homosexual exposure as the mode of transmission (2.8% vs. 6.0% among those not recruited, *p* < 0.001). There were no significant differences in other categories.

### Degree of misclassification

Table 2 shows the prevalence of risk factors within the four registered modes of transmission categories. More than one-third (36.3%) of men registered with heterosexual exposure as the mode of transmission reported injecting drug use risk, 49.1% were HCV positive, and 7.8% reported having sex with men. Table 2 also shows the registered modes of transmission categories that were assigned to people with specific risk factors. For instance, only 37.7% of men reporting sex with men were registered with homosexual exposure as the mode of transmission. This percent ranged from 5.3% in the ≥45-year age category to 66.7% in the < 25 years category. Data disaggregated by region are presented in Supplement Table S4.

**Table 2:** Prevalence of risk factors by registered mode of transmission, sex and age

	Risk factor	Registered mode of transmission									
		HET		IDU		MSM		OTH		Total	
		N	Col %	N	Col %	N	Col %	N	Col %	N	Col %
		Row %		Row %		Row %		Row %		Row %	
Men	Total	609	42.2%	574	75.9%	59	93.7%	11	47.8%	1253	54.8%
		48.6%		45.8%		4.7%		0.9%		100.0%	
	het	590	96.9%	563	98.1%	39	66.1%	11	100.0%	1203	96.0%
		49.0%		46.8%		3.2%		1.0%		100.0%	
	hrh	310	51.2%	411	71.7%	11	18.6%	6	54.5%	738	59.1%
		42.0%		55.7%		1.5%		0.8%		100.0%	
	sti	221	38.2%	239	44.4%	31	53.4%	3	27.3%	494	41.7%
		44.7%		48.4%		6.3%		0.6%		100.0%	
	idu	220	36.3%	503	87.8%	6	10.3%	6	54.5%	735	58.9%
		29.9%		68.4%		0.8%		0.9%		100.0%	
	hcv	293	49.1%	452	80.9%	6	10.7%	4	40.0%	755	61.8%
		38.8%		59.9%		0.8%		0.5%		100.0%	
	hbv	255	42.7%	342	61.3%	27	48.2%	4	40.0%	628	51.4%
		40.6%		54.5%		4.3%		0.6%		100.0%	
	msm	46	7.8%	34	6.1%	49	83.1%	1	9.1%	130	10.6%
		35.4%		26.2%		37.7%		0.7%		100.0%	
	nos	95	15.9%	51	9.2%	7	12.5%	1	9.1%	154	12.7%
		61.7%		33.1%		4.5%		0.7%		100.0%	
	pen	360	64.6%	398	74.8%	21	43.8%	10	90.9%	789	68.7%
		45.6%		50.4%		2.7%		1.3%		100.0%	
Women	Total	834	57.8%	182	24.1%	4	6.3%	12	52.2%	1032	45.2%
		80.8%		17.6%		0.4%		1.2%		100.0%	
	het	824	98.8%	177	97.3%	4	100.0%	11	91.7%	1016	98.4%
		81.1%		17.4%		0.4%		1.1%		100.0%	
	hrh	328	39.5%	129	70.9%	2	50.0%	4	33.3%	463	45.0%
		70.8%		27.9%		0.4%		0.9%		100.0%	
	sti	286	36.3%	83	49.7%	1	50.0%	4	36.4%	374	38.6%
		76.5%		22.2%		0.3%		1.0%		100.0%	
	idu	136	16.5%	138	76.2%	1	25.0%	5	41.7%	280	27.5%
		48.6%		49.3%		0.4%		1.7%		100.0%	
	hcv	301	37.2%	121	68.0%	2	50.0%	2	16.7%	426	42.4%
		70.7%		28.4%		0.5%		0.4%		100.0%	
	hbv	286	35.3%	97	54.5%	2	50.0%	1	8.3%	386	38.4%
		74.1%		25.1%		0.5%		0.3%		100.0%	
	msm	0	0.0%	0	0.0%	0	0.0%	0	0.0%	0	0.0%
		0.0%		0.0%		0.0%		0.0%		0.0%	
	nos	161	19.9%	31	17.4%	1	25.0%	1	10.0%	194	19.3%
		83.0%		16.0%		0.5%		0.5%		100.0%	
	pen	423	54.3%	119	72.1%	3	75.0%	4	40.0%	549	57.3%
		77.0%		21.7%		0.5%		0.8%		100.0%	
<=24	Total	122	8.5%	21	2.8%	14	22.2%	0	0.0%	157	6.9%
		77.7%		13.4%		8.9%		0.0%		100.0%	
	het	120	98.4%	21	100.0%	9	64.3%	0	0.0%	150	95.5%
		80.0%		14.0%		6.0%		0.0%		100.0%	
	hrh	40	33.1%	11	52.4%	2	14.3%	0	0.0%	53	34.0%
		75.5%		20.8%		3.7%		0.0%		100.0%	
	sti	34	30.6%	5	29.4%	7	53.8%	0	0.0%	46	32.6%
		73.9%		10.9%		15.2%		0.0%		100.0%	
	idu	9	7.5%	11	55.0%	0	0.0%	0	0.0%	20	13.1%
		45.0%		55.0%		0.0%		0.0%		100.0%	
	hcv	31	25.6%	10	47.6%	0	0.0%	0	0.0%	41	26.3%
		75.6%		24.4%		0.0%		0.0%		100.0%	
	hbv	31	25.6%	9	42.9%	7	50.0%	0	0.0%	47	30.1%
		66.0%		19.1%		14.9%		0.0%		100.0%	
	msm	6	24.0%	0	0.0%	12	85.7%	0	0.0%	18	39.1%
		33.3%		0.0%		66.7%		0.0%		100.0%	
	nos	19	16.5%	1	4.8%	2	14.3%	0	0.0%	22	14.7%
		86.4%		4.5%		9.1%		0.0%		100.0%	
	pen	58	50.9%	14	66.7%	4	33.3%	0	0.0%	76	51.7%
		76.3%		18.4%		5.3%		0.0%		100.0%	
25–44	Total	1023	70.9%	626	82.8%	45	71.4%	18	78.3%	1712	74.9%
		59.8%		36.6%		2.6%		1.0%		100.0%	
	het	1013	99.0%	618	98.7%	31	68.9%	18	100.0%	1680	98.1%
		60.3%		36.8%		1.8%		1.1%		100.0%	
	hrh	482	47.2%	457	73.1%	10	22.2%	9	50.0%	958	56.1%
		50.3%		47.7%		1.0%		1.0%		100.0%	
	sti	368	37.8%	252	43.2%	22	50.0%	4	23.5%	646	39.9%
		57.0%		39.0%		3.4%		0.6%		100.0%	
	idu	265	26.1%	545	87.1%	6	13.3%	8	44.4%	824	48.4%
		32.2%		66.1%		0.7%		1.0%		100.0%	
	hcv	435	43.6%	489	80.3%	7	16.7%	5	29.4%	936	56.2%
		46.5%		52.2%		0.7%		0.6%		100.0%	
	hbv	383	38.4%	365	60.0%	19	45.2%	2	11.8%	769	46.2%
		49.8%		47.5%		2.5%		0.2%		100.0%	
	msm	26	6.2%	30	6.2%	36	85.7%	1	12.5%	93	9.8%
		28.0%		32.3%		38.7%		1.0%		100.0%	
	nos	163	16.3%	65	10.7%	6	14.0%	1	6.3%	235	14.1%
		69.4%		27.7%		2.6%		0.3%		100.0%	
	pen	569	60.1%	432	74.7%	18	50.0%	11	68.8%	1030	65.4%
		55.2%		41.9%		1.7%		1.2%		100.0%	
> = 45	Total	298	20.7%	109	14.4%	4	6.3%	5	21.7%	416	18.2%
		71.6%		26.2%		1.0%		1.2%		100.0%	
	het	281	94.3%	101	92.7%	3	75.0%	4	80.0%	389	93.5%
		72.2%		26.0%		0.8%		1.0%		100.0%	
	hrh	116	39.5%	72	66.1%	1	25.0%	1	20.0%	190	46.1%
		61.1%		37.9%		0.5%		0.5%		100.0%	
	sti	105	37.4%	65	62.5%	3	100.0%	3	60.0%	176	44.8%
		59.7%		36.9%		1.7%		1.7%		100.0%	
	idu	82	27.8%	85	78.7%	1	25.0%	3	60.0%	171	41.5%
		48.0%		49.7%		0.6%		1.7%		100.0%	
	hcv	128	44.4%	74	69.2%	1	25.0%	1	20.0%	204	50.5%
		62.7%		36.3%		0.5%		0.5%		100.0%	
	hbv	127	44.1%	65	60.7%	3	75.0%	3	60.0%	198	49.0%
		64.1%		32.8%		1.5%		1.6%		100.0%	
	msm	14	9.4%	4	5.6%	1	33.3%	0	0.0%	19	8.4%
		73.7%		21.1%		5.2%		0.0%		100.0%	
	nos	74	25.1%	16	15.4%	0	0.0%	1	20.0%	91	22.4%
		81.3%		17.6%		0.0%		1.1%		100.0%	
	pen	156	56.5%	71	72.4%	2	50.0%	3	60.0%	232	60.6%
		67.2%		30.6%		0.9%		1.3%		100.0%	
Total		1443	100.0%	756	100.0%	63	100.0%	23	100.0%	2285	100.0%
		63.2%		33.1%		2.8%		0.9%		100.0%	
	het	1414	98.0%	740	97.9%	43	68.3%	22	95.7%	2219	97.1%
		63.7%		33.3%		1.9%		1.1%		100.0%	
	hrh	638	44.4%	540	71.5%	13	20.6%	10	43.5%	1201	52.7%
		53.1%		45.0%		1.1%		0.8%		100.0%	
	sti	507	37.1%	322	45.7%	32	53.3%	7	31.8%	868	40.3%
		58.4%		37.1%		3.7%		0.8%		100.0%	
	idu	356	24.9%	641	85.0%	7	11.3%	11	47.8%	1015	44.8%
		35.1%		63.2%		0.7%		1.0%		100.0%	
	hcv	594	42.2%	573	77.7%	8	13.3%	6	27.3%	1181	53.1%
		50.3%		48.5%		0.7%		0.5%		100.0%	
	hbv	541	38.5%	439	59.6%	29	48.3%	5	22.7%	1014	45.6%
		53.4%		43.3%		2.9%		0.4%		100.0%	
	msm	46	7.8%	34	6.1%	49	83.1%	1	9.1%	130	10.6%
		35.4%		26.2%		37.7%		0.7%		100.0%	
	nos	256	18.2%	82	11.2%	8	13.3%	2	9.5%	348	15.7%
		73.6%		23.6%		2.3%		0.5%		100.0%	
	pen	783	58.6%	517	74.2%	24	46.2%	14	66.7%	1338	63.5%
		58.5%		38.6%		1.8%		1.1%		100.0%	

Risk factors (for definitions see Table 1): *het* heterosexual exposure, *hrh* high-risk heterosexual exposure, *sti* sexually transmitted infections, *idu* injecting drug use, *hcv* exposure to HCV, *hbv* exposure to HBV, *msm* homosexual exposure, *nos* nosocomial exposure, *pen* skin penetration exposure

Modes of transmission: *HET* heterosexual, *IDU* injecting drug use, *MSM* homosexual, *OTH* other

Most participants reported exposure to more than one risk factor. Prevalence of two-factor combinations is shown in Table 3. For instance, 76.9% of men who reported having sex with men also had heterosexual exposure, and 25.4% of them reported injecting drug use. Prevalence of anti-HCV antibodies was highest (82.3%) among participants reporting injecting drug use, followed by those with HBV exposure (76.8%), high-risk heterosexual exposure (65.2%), skin penetration risk (56.7%), and STI history (54.7%).

**Table 3:** Prevalence of risk factor combinations

First risk factor	Second risk factor																			
	het		hrh		sti		idu		hcv		hbv		msm		nos		pen		Total N	
	N	Row %	N	Row %	N	Row %	N	Row %	N	Row %	N	Row %	N	Row %	N	Row %	N	Row %	N	Row %
het	**2219**	**100.0%**	1201	54.1%	836	37.7%	989	44.6%	1159	52.2%	981	44.2%	100	4.5%	333	15.0%	1301	58.6%	**2219**	**100.0%**
hrh	1201	100.0%	**1201**	**100.0%**	534	44.5%	775	64.5%	783	65.2%	608	50.6%	45	3.7%	129	10.7%	817	68.0%	**1201**	**100.0%**
sti	836	96.3%	534	61.5%	**868**	**100.0%**	438	50.5%	475	54.7%	439	50.6%	58	6.7%	109	12.6%	601	69.2%	**868**	**100.0%**
idu	989	97.4%	775	76.4%	438	43.2%	**1015**	**100.0%**	835	82.3%	628	61.9%	33	3.3%	98	9.7%	698	68.8%	**1015**	**100.0%**
hcv	1159	98.1%	783	66.3%	475	40.2%	835	70.7%	**1181**	**100.0%**	779	66.0%	37	3.1%	154	13.0%	759	64.3%	**1181**	**100.0%**
hbv	981	96.7%	608	60.0%	439	43.3%	628	61.9%	779	76.8%	**1014**	**100.0%**	60	5.9%	143	14.1%	634	62.5%	**1014**	**100.0%**
msm	100	76.9%	45	34.6%	58	44.6%	33	25.4%	37	28.5%	60	46.2%	**130**	**100.0%**	16	12.3%	72	55.4%	**130**	**100.0%**
nos	333	95.7%	129	37.1%	109	31.3%	98	28.2%	154	44.3%	143	41.1%	16	4.6%	**348**	**100.0%**	225	64.7%	**348**	**100.0%**
pen	1301	97.2%	817	61.1%	601	44.9%	698	52.2%	759	56.7%	634	47.4%	72	5.4%	225	16.8%	**1338**	**100.0%**	**1338**	**100.0%**

The Row % shows the prevalence of second risk factor among patients reporting the first risk factor

Risk factors (for definitions see Table 1): *het* heterosexual exposure, *hrh* high-risk heterosexual exposure, *sti* sexually transmitted infections, *idu* injecting drug use, *hcv* exposure to HCV, *hbv* exposure to HBV, *msm* homosexual exposure, *nos* nosocomial exposure, *pen* skin penetration exposure

Although registered modes of transmission and survey-based modes of transmission correlated (Table 4), the correlation was not perfect. Only 51.4% of patients who registered their mode of transmission as heterosexual were in the same category in our survey. Of the remaining patients with heterosexual exposure as the registered mode of transmission, 45.8% had injecting drug use exposure, and 2.9% (6.9% of men) were MSM who did not inject. The Kappa statistic, presented in Table 5 (with regional data in Supplement Table S5), indicates that the agreement between registered and survey-based results in the majority of categories is fair (0.3–0.4 on the scale from − 1 to 1). Slightly poorer agreement was observed among women, and higher agreement was for the homosexual mode of transmission.

**Table 4:** Cross-tabulation of registered and survey-based mode of transmission by age and sex

	Survey-based mode of transmission	Registered mode of transmission									
		HET		IDU		MSM		OTH		Total	
		N	Col %	N	Col %	N	Col %	N	Col %	N	Col %
		Row %		Row %		Row %		Row %		Row %	
Men	HET	240	39.9%	39	6.8%	5	8.5%	3	27.3%	287	23.0%
		83.6%		13.6%		1.7%		1.1%		100.0%	
	IDU	327	54.3%	530	92.3%	9	15.3%	7	63.6%	873	70.1%
		37.5%		60.7%		1.0%		0.8%		100.0%	
	MSM	35	5.8%	5	0.9%	45	76.3%	1	9.1%	86	6.9%
		40.7%		5.8%		52.3%		1.2%		100.0%	
	UNK	0	0.0%	0	0.0%	0	0.0%	0	0.0%	0	0.0%
		0.0%		0.0%		0.0%		100.0%		0.0%	
Women	HET	498	59.7%	33	18.1%	1	25.0%	6	50.0%	538	52.1%
		92.6%		6.1%		0.2%		1.1%		100.0%	
	IDU	331	39.7%	149	81.9%	3	75.0%	5	41.7%	488	47.3%
		67.8%		30.5%		0.6%		1.1%		100.0%	
	UNK	5	0.6%	0	0.0%	0	0.0%	1	8.3%	6	0.6%
		83.3%		0.0%		0.0%		16.7%		100.0%	
<=24	HET	83	68.6%	6	28.6%	2	14.3%	0	0.0%	91	58.3%
		91.2%		6.6%		2.2%		0.0%		100.0%	
	IDU	32	26.4%	15	71.4%	0	0.0%	0	0.0%	47	30.1%
		68.1%		31.9%		0.0%		0.0%		100.0%	
	MSM	6	5.0%	0	0.0%	12	85.7%	0	0.0%	18	11.5%
		33.3%		0.0%		66.7%		0.0%		100.0%	
	UNK	0	0.0%	0	0.0%	0	0.0%	0	0.0%	0	0.0%
		0.0%		0.0%		0.0%		100.0%		0.0%	
25–44	HET	519	50.8%	52	8.3%	2	4.4%	8	44.4%	581	34.0%
		89.3%		9.0%		0.3%		1.4%		100.0%	
	IDU	482	47.2%	571	91.2%	11	24.4%	9	50.0%	1073	62.7%
		44.9%		53.2%		1.0%		0.9%		100.0%	
	MSM	19	1.9%	3	0.5%	32	71.1%	1	5.6%	55	3.2%
		34.5%		5.5%		58.2%		1.8%		100.0%	
	UNK	1	0.1%	0	0.0%	0	0.0%	0	0.0%	1	0.1%
		100.0%		0.0%		0.0%		0.0%		100.0%	
> = 45	HET	136	46.3%	14	12.8%	2	50.0%	1	20.0%	153	37.1%
		88.9%		9.2%		1.3%		0.6%		100.0%	
	IDU	144	49.0%	93	85.3%	1	25.0%	3	60.0%	241	58.5%
		59.8%		38.6%		0.4%		1.2%		100.0%	
	MSM	10	3.4%	2	1.8%	1	25.0%	0	0.0%	13	3.2%
		76.9%		15.4%		7.7%		0.0%		100.0%	
	UNK	4	1.4%	0	0.0%	0	0.0%	1	20.0%	5	1.2%
		80.0%		0.0%		0.0%		20.0%		100.0%	
**Total**	**HET**	**738**	**51.4%**	**72**	**9.5%**	**6**	**9.5%**	**9**	**39.1%**	**825**	**36.2%**
		**89.5%**		**8.7%**		**0.7%**		**1.1%**		**100.0%**	
	**IDU**	**658**	**45.8%**	**679**	**89.8%**	**12**	**19.0%**	**12**	**52.2%**	**1361**	**59.7%**
		**48.3%**		**49.9%**		**0.9%**		**0.9%**		**100.0%**	
	**MSM**	**35**	**2.4%**	**5**	**0.7%**	**45**	**71.4%**	**1**	**4.3%**	**86**	**3.8%**
		**40.7%**		**5.8%**		**52.3%**		**1.2%**		**100.0%**	
	**UNK**	**5**	**0.3%**	**0**	**0.0%**	**0**	**0.0%**	**1**	**4.3%**	**6**	**0.3%**
		**83.3%**		**0.0%**		**0.0%**		**16.7%**		**100.0%**	

Modes of transmission: *HET* heterosexual, *IDU* injecting drug use, *MSM* homosexual, *OTH* other

**Table 5:** Misclassification of modes of transmission by sex and age

		Registry (verified records)		Survey		McNemar *p*-value	Kappa	Kappa *p*-value	Misclassification	Extrapolation coefficient
		N	MoT %	N	MoT %					
Men							0.33	< 0.001		
	HET	602	48.3%	287	23.0%	< 0.001	0.33	< 0.001	109.8%	47.7%
	IDU	574	46.1%	873	70.1%	< 0.001	0.40	< 0.001	−34.2%	152.1%
	MSM	59	4.7%	86	6.9%	< 0.001	0.60	< 0.001	−31.4%	145.8%
	UNK	11	0.9%	0	0.0%	0.001	0.00	< 0.001	0.0%	0.0%
Women							0.25	< 0.001		
	HET	834	80.8%	538	52.1%	< 0.001	0.25	< 0.001	55.0%	64.5%
	IDU	182	17.6%	488	47.3%	< 0.001	0.25	< 0.001	−62.7%	268.1%
	MSM^a^	4	0.4%	0	0.0%	0.125	0.00	< 0.001	0.0%	0.0%
	UNK	12	1.2%	6	0.6%	0.210	0.10	< 0.001	100.0%	50.0%
<=24							0.35	< 0.001		
	HET	121	77.6%	91	58.3%	< 0.001	0.35	< 0.001	33.0%	75.2%
	IDU	21	13.5%	47	30.1%	< 0.001	0.31	< 0.001	−55.3%	223.8%
	MSM	14	9.0%	18	11.5%	0.289	0.72	< 0.001	−22.2%	128.6%
	UNK	0	0.0%	0	0.0%	< 0.001	0.00	< 0.001	0.0%	0.0%
25–44							0.38	< 0.001		
	HET	1021	59.7%	581	34.0%	< 0.001	0.38	< 0.001	75.7%	56.9%
	IDU	626	36.6%	1073	62.7%	< 0.001	0.39	< 0.001	−41.7%	171.4%
	MSM	45	2.6%	55	3.2%	0.134	0.63	< 0.001	−18.2%	122.2%
	UNK	18	1.1%	1	0.1%	< 0.001	0.00	0.918	1700.0%	5.6%
> = 45							0.23	< 0.001		
	HET	294	71.4%	153	37.1%	< 0.001	0.23	< 0.001	92.2%	52.0%
	IDU	109	26.5%	241	58.5%	< 0.001	0.26	< 0.001	−54.8%	221.1%
	MSM	4	1.0%	13	3.2%	0.035	0.10	0.012	−69.2%	325.0%
	UNK	5	1.2%	5	1.2%	1.000	0.19	< 0.001	0.0%	100.0%
**Grand Total**							**0.37**	**< 0.001**		
	**HET**	**1436**	**63.0%**	**825**	**36.2%**	**< 0.001**	**0.36**	**< 0.001**	**74.1%**	**57.5%**
	**IDU**	**756**	**33.2%**	**1361**	**59.7%**	**< 0.001**	**0.37**	**< 0.001**	**−44.5%**	**180.0%**
	**MSM**	**63**	**2.8%**	**86**	**3.8%**	**0.004**	**0.59**	**< 0.001**	**−26.7%**	**136.5%**
	**UNK**	**23**	**1.0%**	**6**	**0.3%**	**0.002**	**0.07**	**< 0.001**	**283.3%**	**26.1%**

Modes of transmission: *HET* heterosexual, *IDU* injecting drug use, *MSM* homosexual, *OTH* other

^a^Four women had homosexual exposure marked as a mode of transmission in their registration record due to data entry error

The resulting distribution of registered modes of transmission and survey-based modes of transmission among survey participants showed significant differences in all four categories (Table 5). The proportion of cases attributable to injecting drug use was higher in our survey compared to registration records (70.1% vs. 46.2% among men; 47.3% vs. 17.6% among women), the proportion of cases related to homosexual exposure was also higher (6.9% vs. 4.7%), and the number of infections likely acquired through heterosexual transmission was lower (23.0% vs. 48.2% among men; 52.1% vs. 80.7% among women). The degree of misclassification was proportionally greatest among injecting drug users (the true number of cases is underestimated by at least 44.5%), followed by MSM (underestimated by at least 26.7%). Together, this resulted in overestimation of the proportion of heterosexual exposure as the mode of transmission by at least 74.1%. The degree of misclassification varied widely across regions, ranging from + 17.8 to + 121.4% for heterosexual exposure, from − 21.4% to − 65.7% for injecting drug use, and from + 25.0% to − 100.0% for homosexual exposure (Supplement Table S5).

### Extrapolation results

Adjusting for the magnitude of misclassification, we estimated that approximately 43.6% of all patients registered in 2015 in Ukraine had acquired HIV through heterosexual exposure (compared to 70.1% in the official Ministry of Health report), 52.1% had acquired HIV through injecting drug use (compared to 26.7%), 4.2% had acquired HIV through homosexual exposure (compared to 2.9%), and 0.1% through other modes of transmission (compared to 0.3%).

### Trends over time

**Fig. 1:**
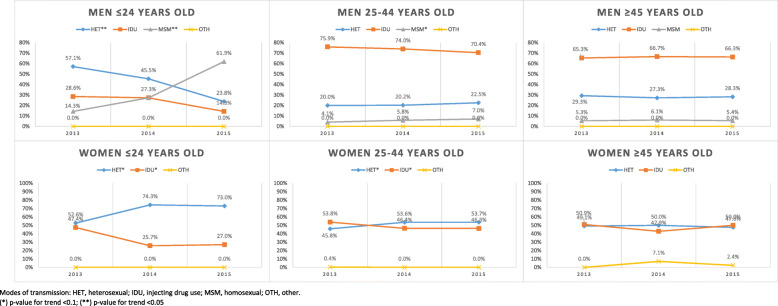
Trends in survey-based mode of transmission by sex and age. Modes of transmission: HET, heterosexual; IDU, injecting drug use; MSM, homosexual; OTH, other. (*) *p*-value for trend < 0.1; (**) *p*-value for trend < 0.05

We assessed the significance of trends in the registered modes of transmission categories, both in the official reports and in the verified registry, in survey-based modes of transmission categories, and in individual risk factors (Tables 6, 7, 8 and 9). The proportion of the three main registered modes of transmission categories (heterosexual exposure, injecting drug use, and homosexual exposure) did not significantly change over time in either the official reports or the registry. The proportion of cases in the “other” category significantly decreased in the official reports (1.1 to 0.5% to 0.4% in 2013, 2014, and 2015 respectively; *p* = 0.019), but this trend was not confirmed in the registry. The proportion of cases likely attributed to homosexual exposure increased significantly according to the survey-based modes of transmission results, from 2.5% (2013) to 3.5% (2014) to 5.2% (2015; *p* = 0.005). This increase was particularly striking in the < 25-year age group with more than 6-time increase over the study period to 23.2% of all men and women in this group in 2015. The increase in cases attributed to homosexual exposure in two cities, Kyiv and Lviv, largely drove the overall trend (Supplement Tables S6, S7, S8 and S9). The proportion of cases attributed to injecting drug use as a survey-based mode of transmission decreased significantly, from 63.2% (2013) to 58.6% (2014) to 57.5% (2015; *p* = 0.022). The proportion of cases attributed to heterosexual transmission was stable at slightly above 37% in 2014 and 2015. Figure 1 displays the trends disaggregated by age and sex.

**Table 6:** Trends in modes of transmission by sex and age in the official reports

		2013	2014	2015	2013	2014	2015	*p*-value for trend
		N	N	N	MoT %	MoT %	MoT %	
Total		1421	1209	1283				0.035
	HET	868	725	808	61.1%	60.0%	63.0%	0.329
	IDU	490	431	409	34.5%	35.6%	31.9%	0.166
	MSM	47	47	61	3.3%	3.9%	4.8%	0.055
	OTH	16	6	5	1.1%	0.5%	0.4%	0.019

Modes of transmission: HET heterosexual, *IDU* injecting drug use, *MSM* homosexual, *OTH* other

**Table 7:** Trends in modes of transmission by sex and age in the verified registry

		2013	2014	2015	2013	2014	2015	*p*-value for trend
		N	N	N	MoT %	MoT %	MoT %	
Males		697	658	741				0.635
	HET	326	321	375	46.8%	48.8%	50.6%	0.146
	IDU	323	283	315	46.3%	43.0%	42.5%	0.146
	MSM	43	47	47	6.2%	7.1%	6.3%	0.905
	OTH	5	7	4	0.7%	1.1%	0.5%	0.686
Females		542	452	537				0.482
	HET	446	377	433	82.3%	83.4%	80.6%	0.481
	IDU	88	67	97	16.2%	14.8%	18.1%	0.420
	MSM	3	3	0	0.6%	0.7%	0.0%	0.147
	OTH	5	5	7	0.9%	1.1%	1.3%	0.551
<=24		94	72	91				0.771
	HET	75	55	67	79.8%	76.4%	73.6%	0.323
	IDU	11	9	13	11.7%	12.5%	14.3%	0.601
	MSM	7	8	11	7.4%	11.1%	12.1%	0.295
	OTH	1	0	0	1.1%	0.0%	0.0%	0.244
25–44		936	853	900				0.595
	HET	548	515	531	58.5%	60.4%	59.0%	0.837
	IDU	347	291	326	37.1%	34.1%	36.2%	0.694
	MSM	35	41	33	3.7%	4.8%	3.7%	0.949
	OTH	6	6	10	.6%	.7%	1.1%	0.266
> = 45		209	185	287				0.206
	HET	149	128	210	71.3%	69.2%	73.2%	0.601
	IDU	53	50	73	25.4%	27.0%	25.4%	0.987
	MSM	4	1	3	1.9%	.5%	1.0%	0.956
	OTH	3	6	1	1.4%	3.2%	.3%	0.236
Total		1239	1110	1278				0.880
	HET	772	698	808	62.3%	62.9%	63.2%	0.635
	IDU	411	350	412	33.2%	31.5%	32.2%	0.621
	MSM	46	50	47	3.7%	4.5%	3.7%	0.956
	OTH	10	12	11	0.8%	1.1%	0.9%	0.892

Modes of transmission: *HET* heterosexual, *IDU* injecting drug use, *MSM* homosexual, *OTH* other

**Table 8:** Trends in modes of transmission by sex and age in the survey

		2013	2014	2015	2013	2014	2015	*p*-value for trend
		N	N	N	MoT %	MoT %	MoT %	
Males		409	369	468				0.024
	HET	94	82	111	23.0%	22.2%	23.7%	0.784
	IDU	296	263	314	72.4%	71.3%	67.1%	0.085
	MSM	19	24	43	4.6%	6.5%	9.2%	0.008
	OTH	0	0	0	0.0%	0.0%	0.0%	
Females		357	312	363				0.264
	HET	168	173	197	47.1%	55.4%	54.3%	0.054
	IDU	188	136	164	52.7%	43.6%	45.2%	0.045
	MSM	0	0	0	0.0%	0.0%	0.0%	
	OTH	1	3	2	0.3%	1.0%	0.6%	0.636
<=24		51	45	56				0.033
	HET	39	38	37	76.5%	84.4%	66.1%	0.923
	IDU	10	4	6	19.6%	8.9%	10.7%	0.025
	MSM	2	3	13	3.9%	6.7%	23.2%	0.002
	OTH	0	0	0	0.0%	0.0%	0.0%	
25–44		583	523	594				0.154
	HET	272	254	286	46.7%	48.6%	48.1%	0.200
	IDU	296	251	277	50.8%	48.0%	46.6%	0.059
	MSM	14	18	31	2.4%	3.4%	5.2%	0.058
	OTH	1	0	0	.2%	0.0%	0.0%	0.225
> = 45		129	108	171				0.497
	HET	75	52	89	58.1%	48.1%	52.0%	0.968
	IDU	50	49	72	38.8%	45.4%	42.1%	0.931
	MSM	4	4	7	3.1%	3.7%	4.1%	0.898
	OTH	0	3	3	0.0%	2.8%	1.8%	0.440
Total		766	681	831				0.020
	HET	262	255	308	34.2%	37.4%	37.1%	0.242
	IDU	484	399	478	63.2%	58.6%	57.5%	0.022
	MSM	19	24	43	2.5%	3.5%	5.2%	0.005
	OTH	1	3	2	0.1%	0.4%	0.2%	0.686

Modes of transmission: *HET* heterosexual, *IDU* injecting drug use, *MSM* homosexual, *OTH* other

**Table 9:** Trends in risk factors by sex and age in the survey

		2013	2014	2015	2013	2014	2015	*p*-value for trend
		N	N	N	%	%	%	
Men		410	373	470				
	het	401	360	442	97.8%	96.5%	94.0%	0.004
	hrh	251	213	274	61.4%	57.4%	58.4%	0.476
	sti	154	143	197	40.3%	39.7%	44.5%	0.183
	idu	246	226	263	60.0%	60.9%	56.3%	0.264
	hcv	250	232	273	61.4%	65.4%	59.3%	0.595
	hbv	206	183	239	50.6%	51.5%	52.1%	0.579
	msm	26	38	66	6.4%	10.5%	14.5%	< 0.001
	nos	29	58	67	7.4%	15.8%	14.7%	0.002
	pen	262	231	296	70.1%	67.3%	68.7%	0.787
Women		357	312	363				
	het	356	306	354	99.7%	98.1%	97.5%	0.016
	hrh	174	145	144	48.7%	46.9%	39.8%	0.020
	sti	122	106	146	37.5%	35.1%	42.8%	0.130
	idu	110	78	92	31.1%	25.2%	25.8%	0.119
	hcv	167	122	137	47.4%	41.8%	38.1%	0.023
	hbv	147	110	129	41.8%	37.7%	35.8%	0.151
	msm	0	0	0	0.0%	0.0%	0.0%	
	nos	51	66	77	14.7%	21.7%	21.9%	0.022
	pen	198	153	198	60.0%	53.3%	58.1%	0.812
<=24		52	46	59				
	het	52	45	53	100.0%	97.8%	89.8%	0.008
	hrh	26	11	16	50.0%	23.9%	27.6%	0.029
	sti	14	13	19	31.8%	30.2%	35.2%	0.562
	idu	10	4	6	19.6%	8.9%	10.5%	0.184
	hcv	20	11	10	38.5%	24.4%	16.9%	0.024
	hbv	17	11	19	32.7%	24.4%	32.2%	0.757
	msm	2	3	13	14.3%	27.3%	61.9%	0.004
	nos	2	11	9	4.0%	25.0%	16.1%	0.119
	pen	25	16	35	51.0%	38.1%	62.5%	0.141
25–44		585	528	599				
	het	578	518	584	98.8%	98.1%	97.5%	0.089
	hrh	338	293	327	57.9%	55.6%	54.7%	0.312
	sti	209	193	244	38.5%	37.7%	43.3%	0.090
	idu	296	251	277	50.7%	47.8%	46.6%	0.156
	hcv	335	289	312	58.0%	58.0%	52.9%	0.097
	hbv	273	227	269	47.2%	45.6%	45.7%	0.693
	msm	20	29	44	6.3%	10.1%	12.6%	0.006
	nos	55	88	92	9.8%	17.0%	15.8%	0.004
	pen	364	310	356	67.8%	63.4%	64.7%	0.386
> = 45		130	111	175				
	het	127	103	159	97.7%	92.8%	90.9%	0.019
	hrh	61	54	75	46.9%	50.5%	42.9%	0.442
	sti	53	43	80	44.2%	40.2%	48.2%	0.412
	idu	50	49	72	38.8%	44.1%	41.9%	0.605
	hcv	62	54	88	48.1%	51.9%	51.5%	0.412
	hbv	63	55	80	48.8%	52.9%	46.8%	0.690
	msm	4	6	9	5.4%	9.2%	10.3%	0.364
	nos	23	25	43	18.3%	22.5%	25.3%	0.157
	pen	71	58	103	60.2%	58.6%	62.0%	0.642
Total		767	685	833				
	het	757	666	796	98.7%	97.2%	95.6%	< 0.001
	hrh	425	358	418	55.5%	52.6%	50.3%	0.057
	sti	276	249	343	39.0%	37.6%	43.8%	0.042
	idu	356	304	355	46.6%	44.6%	43.1%	0.172
	hcv	417	354	410	54.9%	54.7%	50.0%	0.092
	hbv	353	293	368	46.5%	45.3%	44.9%	0.692
	msm	26	38	66	6.4%	10.5%	14.5%	<0.001
	nos	80	124	144	10.8%	18.5%	17.8%	<0.001
	pen	460	384	494	65.3%	61.0%	64.0%	0.819

Risk factors (for definitions see Table 1): *het* heterosexual exposure, *hrh* high-risk heterosexual exposure, *sti* sexually transmitted infections, *idu* injecting drug use, *hcv* exposure to HCV, *hbv* exposure to HBV, *msm* homosexual exposure, *nos* nosocomial exposure, *pen* skin penetration exposure

Several individual risk factors changed significantly from 2013 to 2015. The prevalence of heterosexual exposure decreased modestly but significantly, from 98.7% (2013) to 97.2% (2014) to 95.6% (2015; *p* < 0.001), whereas high-risk heterosexual exposure decreased steeply, from 55.5% (2013) to 52.6% (2014) to 50.3% (2015), but this trend did not reach significance (*p* = 0.057). Self-reported injecting drug use risk decreased insignificantly among men from 60.0% (2013) to 60.9% (2014) to 56.3% (2015) and among women from 31.1% (2013) to 25.2% (2014) to 25.8% (2015). HCV seropositivity fluctuated among men from 61.4% (2013) to 65.4% (2014) to 59.3% (2015) and decreased significantly among women from 47.4% (2013) to 41.8% (2014) to 38.1% (2015; *p* = 0.023). The proportion of men reporting sex with other men more than doubled, from 6.4% (2013) to 10.5% (2014) to 14.5% (2015; *p* < 0.001). This increase was particularly obvious among men younger than 25 years: from 14.3% (2013) to 27.3% (2014) to 61.9% (2015; *p* = 0.004). Nosocomial exposure increased from 10.8% (2013) to 18.5% (2014) to 17.8% (2015; *p* < 0.001). HBV exposure and skin penetration factors did not change significantly in men or women.

### Accuracy of reporting

As shown in Supplement Table S3, the distribution of the four registered modes of transmission in the official reports and in the verified registry did not significantly differ. With the exception of Lviv, where not all registration forms were available for verification, the registered modes of transmission did not significantly differ at the regional level.

## Discussion

In this study among patients registered with a diagnosis of HIV infection in 7 regions of Ukraine, we undertook a standardized ascertainment of risk factor information that was designed to be more sensitive through simple efforts to develop enhanced rapport and use of biological markers. Our results demonstrate that standard case registration procedures in HIV clinics in Ukraine are less sensitive in detecting stigmatized behaviors, such as homosexual exposure and injecting drug use, which leads to underestimation of the proportion of cases attributable to these modes of transmission. About two-thirds of patients who self-reported injecting drug use exposure and slightly less than half of those with HCV markers had injecting drug use as the registered mode of transmission. Only about half of men reporting sex with men (who did not inject drugs) had homosexual exposure as the registered mode of transmission. Consequently, the proportion of heterosexual transmission was overestimated by almost 75%. Our findings suggest that Cakalo et al. [8] overestimated the proportion of MSM among men reported as heterosexual (8.2% compared to 5.8% in our data, see Table 4) and underestimated the proportion of PWID (34.5% compared to 54.3% in our data).

After adjusting for misclassification, the national distribution of homosexual exposure as a mode of transmission among HIV cases registered in Ukraine in 2015 was 4.2%, which is close to the average of 4.0% in Eastern European countries [5]. The estimated 52.1% of transmission via injecting drug use in Ukraine remains higher than the average of 26% in the rest of Eastern Europe (excluding Russia, where injecting drug use still accounts for more than half of new HIV infections). Heterosexual transmission of HIV, therefore, is lower than in other Eastern European countries and, as other analyses show, in many cases occurs among partners of PWID [10]. Our findings suggest that the HIV epidemic in Ukraine remains significantly driven by injecting drug use.

We investigated the trends in HIV transmission in seven regions in Ukraine over 3 years (2013–2015). The prevalence of injecting drug use exposure remained high overall but significantly decreased among women and participants younger than 25 years. This finding may suggest a shift from injecting drug use to heterosexual transmission, which accounted for over half of cases among female participants in 2015.

The number and proportion of HIV cases attributable to homosexual exposure more than doubled between 2013 and 2015. Among men younger than 25 years, the proportion of cases attributable to homosexual exposure increased more than six times, from 14.3 to 61.9%. This sharp increase largely occurred in two regions, Kyiv and Lviv, which may indicate an ongoing outbreak, especially among young MSM. Other evidence supports this possibility, including a high HIV incidence rate estimated from LAg assay testing of specimens from the 2013 MSM integrated bio-behavioral survey (IBBS) [19] and increased HIV prevalence in younger MSM according to the 2015 and 2013 IBBS surveys [12]. Importantly, this trend was not significant in the official reports and verified registration records owing to the substantial degree of misclassification. Given the relatively low proportion of MSM cases overall, misclassification may obscure potential outbreaks and delay the public health response.

### Limitations

Our study has several limitations. First, we relied on self-report of risk behaviors, which is prone to recall bias and deliberate underreporting of stigmatized behaviors. To mitigate this limitation, we tested for biological markers and assumed 100% link between the positive anti-HCV results and injecting drug use. On the other hand, prevalence of HCV in the general population in Ukraine may be substantial [20], which may have led to overestimation of the number of injecting drug use-related cases in our study.

Another important limitation of self-reported HIV risk is the uncertainty about the specific behavior that actually led to transmission. Nearly all participants reported heterosexual activity, and about two-thirds reported skin penetration exposure. Thus, there is a possibility that homosexual exposure or injecting drug use were not the actual causes of HIV transmission in these patients. We suggested a hierarchy of most probable modes of transmission based on the probability of transmission per act [16] and the prevalence of infection in respective populations in Ukraine [21, 22], This hierarchy, we believe, has the least bias in the current epidemiological context. A study of risk networks with virus genotyping is needed to establish the probability of acquiring HIV through specific modes when multiple exposures are present.

In our study sample, the number of patients with homosexual exposure as the registered mode of transmission was disproportionately lower than among registered patients who did not participate in our study; therefore the observed prevalence of homosexual exposure in our sample likely underestimates the true level. However, this should not bias our estimate of the degree of misclassification and hence the estimated proportion of HIV infections attributable to homosexual exposure among all patients nationally.

## Conclusion

There is a significant degree of misclassification of key modes of transmission in the case registration system in Ukraine. Improvements in HIV case registration systems, such as more structured and sensitive ascertainment of risk factors, are needed to more accurately assess the epidemic trends and guide programmatic response in Ukraine and other countries where injecting drug use and homosexuality are stigmatized.

We found that HIV transmission via injecting drug use is still high, particularly among men, although this mode of transmission is decreasing significantly. We also found an explosive increase, more than double overall and more than six times in patients younger than 25 years in only 2 years, of HIV infections attributed to homosexual exposure, which correlates with other data [12, 19]. In a resource-limited context, both key populations would benefit from combination prevention, including traditional harm reduction and medication-assisted treatment for opioid users as well as novel interventions such as pre-exposure prophylaxis and the *Test and Treat* approach. Targeting these key populations (MSM and PWID) can help Ukraine achieve ambitious targets set by the World Health Organization European Member States in September 2016 [23] and prevent outbreaks that may occur due to reduction of harm reduction activities [24–28].

## Supplementary information

**Additional file 1: Supplementary file 1.** Participant questionnaire. This is the original version of the study questionnaire in Ukrainian with machine-translated English version.

**Additional file 2: Table S1.** Site selection. **Table S2.** Study population and sample. **Table S3.** Distribution of registered modes of transmission in the official reports, verified registry and study sample. **Table S4.** Prevalence of risk factors by registered mode of transmission and region. **Table S5.** Misclassification of modes of transmission by region. **Table S6.** Trends in modes of transmission by region in the official reports. **Table S7.** Trends in modes of transmission by region in the verified registry. **Table S8.** Trends in modes of transmission by region in the survey. **Table S9.** Trends in risk factors by region in the survey.

## Data Availability

The de-personalized datasets used in the current study are available from the corresponding author on reasonable request. Summary tables with site-level data are presented as Supplements. The study questionnaire is provided in Supplementary Files.

## Abbreviations

AIDS: Acquired immunodeficiency syndrome
CDC: US Centers for disease control and prevention
HBV: Hepatitis B virus
HCV: Hepatitis C virus
HIV: Human immunodeficiency virus
MSM: Men who have sex with men
PWID: People who inject drugs
