# Supplementary material for: Improved ascertainment of modes of HIV transmission in Ukraine indicates importance of drug injecting and homosexual risk
Source: BMC Public Health. 2020 Aug 26;20:1288. doi: 10.1186/s12889-020-09373-2 (PMC7449084; doi:10.1186/s12889-020-09373-2)
Supplement: Supplementary file 2 — Additional file 2: Table S1. Site selection. Table S2. Study population and sample. Table S3. Distribution of registered modes of transmission in the official reports, verified registry and study sample. Table S4. Prevalence of risk factors by registered mode of transmission and region. Table S5. Misclassification of modes of transmission by region. Table S6. Trends in modes of transmission by region in the official reports. Table S7. Trends in modes of transmission by region in the verified registry. Table S8. Trends in modes of transmission by region in the survey. Table S9. Trends in risk factors by region in the survey. [file 12889_2020_9373_MOESM2_ESM.docx]

# Table S1. Site selection.

|  | Region | Total population number in 2010 | Number of HIV cases diagnosed in 2013 | Proportiol contribution in total number of patients diagnosed in 2013 (%) | Number of patients to be recruited for each year |
| --- | --- | --- | --- | --- | --- |
| 1 | Donetsk | 4 466 700 | 3 648 |  |  |
| 2 | Dnipropetrovsk | 3 355 500 | 3 450 | 53 | 411 |
| 3 | Odesa | 2 391 000 | 2 751 |  |  |
| 4 | Mykolaiv | 1 189 500 | 1 081 |  |  |
| 5 | AR Crimea | 1 965 300 | 1 087 |  |  |
| 6 | City of Kyiv | 2 785 100 | 1 405 | 21.6 | 167 |
| 7 | Kyiv | 1 721 800 | 727 |  |  |
| 8 | Luhansk | 2 311 600 | 910 |  |  |
| 9 | Kherson | 1 093 400 | 557 |  |  |
| 10 | Kharkiv | 2 769 100 | 646 | 9.9 | 77 |
| 11 | Zaporozhe | 1 811 700 | 605 |  |  |
| 12 | Cherkasy | 1 295 200 | 456 |  |  |
| 13 | Poltava | 1 499 600 | 508 |  |  |
| 14 | Lviv | 2 549 600 | 418 | 6.4 | 50 |
| 15 | Zhitomir | 1 285 800 | 423 |  |  |
| 16 | Vinnitsa | 1 650 600 | 313 |  |  |
| 17 | Chernihiv | 1 109 700 | 510 |  |  |
| 18 | Kirovograd | 1 017 800 | 345 | 5.3 | 41 |
| 19 | Khmelnytskyi | 1 334 000 | 334 |  |  |
| 20 | Sevastopol | 380 500 | 250 |  |  |
| 21 | Volyn | 1 036 700 | 267 |  |  |
| 22 | Chernivtsi | 904 400 | 108 | 1.7 | 13 |
| 23 | Rivne | 1 151 600 | 254 |  |  |
| 24 | Sumy | 1 172 300 | 206 |  |  |
| 25 | Ivano-Frankivsk | 1 380 700 | 159 |  |  |
| 26 | Ternopil | 1 088 900 | 132 | 2 | 16 |
| 27 | Zakarpattia | 1 244 800 | 81 |  |  |
|  | **Total** | **45 962 900** | **21 631** | **100** | **775** |

# Table S2. Study population and sample.

| Region | Reports | Registry (verified records) | | | | | | | | | | | | | | |
| --- | --- | --- | --- | --- | --- | --- | --- | --- | --- | --- | --- | --- | --- | --- | --- | --- |
|  | Total N | Total N | Deceased | | Migrated | | Incarcerated | | Lost to follow-up | | Not contacted | | Declined to participate | | Participated in the survey | |
|  |  |  | N | % | N | % | N | % | N | % | N | % | N | % | N | % |
| Dnipro | 1936 | 1668 | 109 | 6.5% | 11 | 0.7% | 7 | 0.4% | 8 | 0.5% | 229 | 13.7% | 72 | 4.3% | 1232 | 73.9% |
| Kyiv | 879 | 892 | 94 | 10.5% | 21 | 2.4% | 2 | 0.2% | 198 | 22.2% | 1 | 0.1% | 130 | 14.6% | 446 | 50.0% |
| Kropivnitsky | 285 | 284 | 64 | 22.5% | 4 | 1.4% | 1 | 0.4% | 24 | 8.5% | 2 | 0.7% | 30 | 10.6% | 159 | 56.0% |
| Lviv | 321 | 271 | 23 | 8.5% | 5 | 1.8% | 63 | 23.2% | 11 | 4.1% | 3 | 1.1% | 9 | 3.3% | 157 | 57.9% |
| Ternopil | 88 | 108 | 2 | 1.9% | 1 | 0.9% | 0 | 0.0% | 1 | 0.9% | 0 | 0.0% | 9 | 8.3% | 95 | 88.0% |
| Kharkiv | 353 | 344 | 34 | 9.9% | 7 | 2.0% | 2 | 0.6% | 91 | 26.5% | 22 | 6.4% | 28 | 8.1% | 160 | 46.5% |
| Chernivtsi | 51 | 60 | 7 | 11.7% | 6 | 10.0% | 1 | 1.7% | 6 | 10.0% | 0 | 0.0% | 4 | 6.7% | 36 | 60.0% |
| **Grand Total** | **3913** | **3627** | **333** | **9.2%** | **55** | **1.5%** | **76** | **2.1%** | **339** | **9.3%** | **257** | **7.1%** | **282** | **7.8%** | **2285** | **63.0%** |

# Table S3. Distribution of registered modes of transmission in the official reports, verified registry and study sample

| City | Official reports | | Registry (verified records) | | | | | p-Values for difference | |
| --- | --- | --- | --- | --- | --- | --- | --- | --- | --- |
|  |  |  | Not surveyed | | Surveyed | |  | Between surveyed and not surveyed | Between registry and official reports |
|  | N | MoT % | N | MoT % | N | MoT % | Surveyed % |  |  |
| **Dnipro (total)** | **1936** |  | **436** |  | **1232** |  | **73.9%** | **0.911** | **0.455** |
| HET | 1244 | 64.3% | 283 | 64.9% | 799 | 64.9% | 73.8% | 0.984 | 0.702 |
| IDU | 668 | 34.5% | 147 | 33.7% | 410 | 33.3% | 73.6% | 0.868 | 0.483 |
| MSM | 8 | 0.4% | 3 | 0.7% | 10 | 0.8% | 76.9% | 0.546 | 0.150 |
| UNK | 16 | 0.8% | 3 | 0.7% | 13 | 1.1% | 81.3% | 0.364 | 0.672 |
| **Kyiv (total)** | **879** |  | **446** |  | **446** |  | **50.0%** | **<0.001** | **0.896** |
| HET | 460 | 52.3% | 276 | 61.9% | 193 | 43.3% | 41.2% | <0.001 | 0.917 |
| IDU | 301 | 34.2% | 96 | 21.5% | 206 | 46.2% | 68.2% | <0.001 | 0.864 |
| MSM | 113 | 12.9% | 72 | 16.1% | 46 | 10.3% | 39.0% | 0.100 | 0.816 |
| UNK | 5 | 0.6% | 2 | 0.4% | 1 | 0.2% | 33.3% | 0.500 | 0.355 |
| Kropivnitsky (total) | 285 |  | 125 |  | 159 |  | 56.0% | 0.525 | 0.604 |
| HET | 222 | 77.9% | 96 | 76.8% | 124 | 78.0% | 56.4% | 0.812 | 0.902 |
| IDU | 63 | 22.1% | 28 | 22.4% | 35 | 22.0% | 55.6% | 0.938 | 0.982 |
| MSM | 0 | 0.0% | 0 | 0.0% | 0 | 0.0% |  |  |  |
| UNK | 0 | 0.0% | 1 | 0.8% | 0 | 0.0% | 0.0% | 0.440 | 0.499 |
| Lviv (total) | 321 |  | 114 |  | 157 |  | 57.9% | 0.046 | <0.001 |
| HET | 176 | 54.8% | 79 | 69.3% | 123 | 78.3% | 60.9% | 0.092 | <0.001 |
| IDU | 121 | 37.7% | 35 | 30.7% | 31 | 19.7% | 47.0% | 0.038 | 0.001 |
| MSM | 24 | 7.5% | 0 | 0.0% | 0 | 0.0% |  |  | <0.001 |
| UNK | 0 | 0.0% | 0 | 0.0% | 3 | 1.9% | 100.0% | 0.193 | 0.095 |
| Ternopil (total) | 88 |  | 13 |  | 95 |  | 88.0% | 0.008 | 0.279 |
| HET | 63 | 71.6% | 11 | 84.6% | 71 | 74.7% | 86.6% | 0.347 | 0.491 |
| IDU | 23 | 26.1% | 0 | 0.0% | 22 | 23.2% | 100.0% | 0.042 | 0.340 |
| MSM | 0 | 0.0% | 2 | 15.4% | 1 | 1.1% | 33.3% | 0.038 | 0.165 |
| UNK | 2 | 2.3% | 0 | 0.0% | 1 | 1.1% | 100.0% | 0.271 | 0.423 |
| Kharkiv (total) | 353 |  | 184 |  | 160 |  | 46.5% | <0.001 | 0.428 |
| HET | 193 | 54.7% | 71 | 38.6% | 106 | 66.3% | 59.9% | <0.001 | 0.394 |
| IDU | 146 | 41.4% | 107 | 58.2% | 43 | 26.9% | 28.7% | <0.001 | 0.549 |
| MSM | 10 | 2.8% | 2 | 1.1% | 6 | 3.8% | 75.0% | 0.101 | 0.673 |
| UNK | 4 | 1.1% | 4 | 2.2% | 5 | 3.1% | 55.6% | 0.414 | 0.148 |
| Chernivtsi (total) | 51 |  | 24 |  | 36 |  | 60.0% | 0.369 | 0.456 |
| HET | 43 | 84.3% | 19 | 79.2% | 27 | 75.0% | 58.7% | 0.709 | 0.314 |
| IDU | 8 | 15.7% | 4 | 16.7% | 9 | 25.0% | 69.2% | <0.001 | 0.423 |
| MSM | 0 | 0.0% | 1 | 4.2% | 0 | 0.0% | 0.0% | 0.443 | 0.541 |
| UNK | 0 | 0.0% | 0 | 0.0% | 0 | 0.0% |  |  |  |
| **Grand Total** | **3913** |  | **1342** |  | **2285** |  | **63.0%** | **<0.001** | **0.345** |
| **HET** | **2401** | **61.4%** | **835** | **62.2%** | **1443** | **63.2%** | **63.3%** | **0.576** | **0.196** |
| **IDU** | **1330** | **34.0%** | **417** | **31.1%** | **756** | **33.1%** | **64.5%** | **0.211** | **0.129** |
| **MSM** | **155** | **4.0%** | **80** | **6.0%** | **63** | **2.8%** | **44.1%** | **<0.001** | **0.967** |
| **UNK** | **27** | **0.7%** | **10** | **0.7%** | **23** | **1.0%** | **69.7%** | **0.423** | **0.283** |

Modes of transmission: HET – heterosexual; IDU – injecting drug use; MSM – homosexual; OTH – other.

# Table S4. Prevalence of risk factors by registered mode of transmission and region.

|  | Risk factor | Registered mode of transmission | | | | | | | | | |
| --- | --- | --- | --- | --- | --- | --- | --- | --- | --- | --- | --- |
|  |  | HET | | IDU | | MSM | | OTH | | Total | |
|  |  | N | Col % | N | Col % | N | Col % | N | Col % | N | Col % |
|  |  | Row % |  | Row % |  | Row % |  | Row % |  | Row % |  |
| Dnipro | Total | 799 | 55.4% | 410 | 54.2% | 10 | 15.9% | 13 | 56.5% | 1232 | 53.9% |
|  |  | 64.9% |  | 33.3% |  | 0.8% |  | 1.0% |  | 100.0% |  |
|  | het | 785 | 98.2% | 394 | 96.1% | 10 | 100.0% | 12 | 92.3% | 1201 | 97.5% |
|  |  | 65.4% |  | 32.8% |  | 0.8% |  | 1.0% |  | 100.0% |  |
|  | hrh | 385 | 48.5% | 286 | 69.9% | 7 | 70.0% | 7 | 53.8% | 685 | 55.9% |
|  |  | 56.2% |  | 41.8% |  | 1.0% |  | 1.0% |  | 100.0% |  |
|  | sti | 345 | 46.4% | 184 | 50.5% | 4 | 50.0% | 5 | 41.7% | 538 | 47.7% |
|  |  | 64.1% |  | 34.2% |  | 0.7% |  | 1.0% |  | 100.0% |  |
|  | idu | 240 | 30.4% | 335 | 81.9% | 6 | 60.0% | 8 | 61.5% | 589 | 48.2% |
|  |  | 40.7% |  | 56.9% |  | 1.0% |  | 1.4% |  | 100.0% |  |
|  | hcv | 377 | 47.3% | 281 | 68.9% | 5 | 50.0% | 5 | 38.5% | 668 | 54.4% |
|  |  | 56.4% |  | 42.1% |  | 0.7% |  | 0.8% |  | 100.0% |  |
|  | hbv | 340 | 42.7% | 212 | 52.0% | 4 | 40.0% | 3 | 23.1% | 559 | 45.5% |
|  |  | 60.8% |  | 37.9% |  | 0.7% |  | 0.6% |  | 100.0% |  |
|  | msm | 12 | 3.6% | 17 | 5.6% | 1 | 16.7% | 0 | 0.0% | 30 | 4.7% |
|  |  | 40.0% |  | 56.7% |  | 3.3% |  | 0.0% |  | 100.0% |  |
|  | nos | 152 | 19.6% | 44 | 11.1% | 1 | 10.0% | 1 | 9.1% | 198 | 16.6% |
|  |  | 76.8% |  | 22.2% |  | 0.5% |  | 0.5% |  | 100.0% |  |
|  | pen | 491 | 65.3% | 295 | 77.8% | 5 | 55.6% | 8 | 72.7% | 799 | 69.4% |
|  |  | 61.5% |  | 36.9% |  | 0.6% |  | 1.0% |  | 100.0% |  |
| Kyiv | Total | 193 | 13.4% | 206 | 27.2% | 46 | 73.0% | 1 | 4.3% | 446 | 19.5% |
|  |  | 43.3% |  | 46.2% |  | 10.3% |  | 0.2% |  | 100.0% |  |
|  | het | 191 | 99.0% | 206 | 100.0% | 27 | 58.7% | 1 | 100.0% | 425 | 95.3% |
|  |  | 44.9% |  | 48.5% |  | 6.4% |  | 0.2% |  | 100.0% |  |
|  | hrh | 94 | 48.7% | 164 | 79.6% | 4 | 8.7% | 0 | 0.0% | 262 | 58.7% |
|  |  | 35.9% |  | 62.6% |  | 1.5% |  | 0.0% |  | 100.0% |  |
|  | sti | 77 | 41.2% | 95 | 46.6% | 25 | 54.3% | 1 | 100.0% | 198 | 45.2% |
|  |  | 38.9% |  | 48.0% |  | 12.6% |  | 0.5% |  | 100.0% |  |
|  | idu | 25 | 13.0% | 198 | 96.1% | 0 | 0.0% | 0 | 0.0% | 223 | 50.0% |
|  |  | 11.2% |  | 88.8% |  | 0.0% |  | 0.0% |  | 100.0% |  |
|  | hcv | 52 | 31.1% | 181 | 94.8% | 2 | 4.7% | 0 | 0.0% | 235 | 58.5% |
|  |  | 22.1% |  | 77.0% |  | 0.9% |  | 0.0% |  | 100.0% |  |
|  | hbv | 51 | 30.5% | 138 | 72.6% | 22 | 51.2% | 1 | 100.0% | 212 | 52.9% |
|  |  | 24.1% |  | 65.1% |  | 10.4% |  | 0.4% |  | 100.0% |  |
|  | msm | 8 | 13.3% | 8 | 5.3% | 44 | 95.7% | 0 | 0.0% | 60 | 23.3% |
|  |  | 13.3% |  | 13.3% |  | 73.3% |  | 0.1% |  | 100.0% |  |
|  | nos | 32 | 17.0% | 20 | 10.1% | 6 | 14.0% | 0 | 0.0% | 58 | 13.5% |
|  |  | 55.2% |  | 34.5% |  | 10.3% |  | 0.0% |  | 100.0% |  |
|  | pen | 113 | 70.2% | 151 | 80.7% | 17 | 43.6% | 1 | 100.0% | 282 | 72.7% |
|  |  | 40.1% |  | 53.5% |  | 6.0% |  | 0.4% |  | 100.0% |  |
| Kropivnitsky | Total | 124 | 8.6% | 35 | 4.6% | 0 | 0.0% | 0 | 0.0% | 159 | 7.0% |
|  |  | 78.0% |  | 22.0% |  | 0.0% |  | 0.0% |  | 100.0% |  |
|  | het | 122 | 98.4% | 35 | 100.0% | 0 | 0.0% | 0 | 0.0% | 157 | 98.7% |
|  |  | 77.7% |  | 22.3% |  | 0.0% |  | 0.0% |  | 100.0% |  |
|  | hrh | 66 | 53.2% | 25 | 71.4% | 0 | 0.0% | 0 | 0.0% | 91 | 57.2% |
|  |  | 72.5% |  | 27.5% |  | 0.0% |  | 0.0% |  | 100.0% |  |
|  | sti | 22 | 18.3% | 11 | 31.4% | 0 | 0.0% | 0 | 0.0% | 33 | 21.3% |
|  |  | 66.7% |  | 33.3% |  | 0.0% |  | 0.0% |  | 100.0% |  |
|  | idu | 45 | 36.6% | 30 | 85.7% | 0 | 0.0% | 0 | 0.0% | 75 | 47.5% |
|  |  | 60.0% |  | 40.0% |  | 0.0% |  | 0.0% |  | 100.0% |  |
|  | hcv | 65 | 52.8% | 30 | 88.2% | 0 | 0.0% | 0 | 0.0% | 95 | 60.5% |
|  |  | 68.4% |  | 31.6% |  | 0.0% |  | 0.0% |  | 100.0% |  |
|  | hbv | 57 | 46.3% | 24 | 70.6% | 0 | 0.0% | 0 | 0.0% | 81 | 51.6% |
|  |  | 70.4% |  | 29.6% |  | 0.0% |  | 0.0% |  | 100.0% |  |
|  | msm | 2 | 3.7% | 4 | 13.3% | 0 | 0.0% | 0 | 0.0% | 6 | 7.1% |
|  |  | 33.3% |  | 66.7% |  | 0.0% |  | 0.0% |  | 100.0% |  |
|  | nos | 29 | 24.0% | 6 | 17.1% | 0 | 0.0% | 0 | 0.0% | 35 | 22.4% |
|  |  | 82.9% |  | 17.1% |  | 0.0% |  | 0.0% |  | 100.0% |  |
|  | pen | 60 | 51.7% | 20 | 62.5% | 0 | 0.0% | 0 | 0.0% | 80 | 54.1% |
|  |  | 75.0% |  | 25.0% |  | 0.0% |  | 0.0% |  | 100.0% |  |
| Lviv | Total | 123 | 8.5% | 31 | 4.1% | 0 | 0.0% | 3 | 13.0% | 157 | 6.9% |
|  |  | 78.3% |  | 19.7% |  | 0.0% |  | 2.0% |  | 100.0% |  |
|  | het | 118 | 95.9% | 31 | 100.0% | 0 | 0.0% | 3 | 100.0% | 152 | 96.8% |
|  |  | 77.6% |  | 20.4% |  | 0.0% |  | 2.0% |  | 100.0% |  |
|  | hrh | 38 | 31.1% | 14 | 45.2% | 0 | 0.0% | 1 | 33.3% | 53 | 34.0% |
|  |  | 71.7% |  | 26.4% |  | 0.0% |  | 1.9% |  | 100.0% |  |
|  | sti | 21 | 18.6% | 5 | 16.7% | 0 | 0.0% | 0 | 0.0% | 26 | 17.8% |
|  |  | 80.8% |  | 19.2% |  | 0.0% |  | 0.0% |  | 100.0% |  |
|  | idu | 35 | 28.7% | 14 | 46.7% | 0 | 0.0% | 2 | 66.7% | 51 | 32.9% |
|  |  | 68.6% |  | 27.5% |  | 0.0% |  | 3.9% |  | 100.0% |  |
|  | hcv | 52 | 44.1% | 17 | 56.7% | 0 | 0.0% | 1 | 50.0% | 70 | 46.7% |
|  |  | 74.3% |  | 24.3% |  | 0.0% |  | 1.4% |  | 100.0% |  |
|  | hbv | 47 | 39.8% | 17 | 56.7% | 0 | 0.0% | 0 | 0.0% | 64 | 42.7% |
|  |  | 73.4% |  | 26.6% |  | 0.0% |  | 0.0% |  | 100.0% |  |
|  | msm | 11 | 19.3% | 4 | 17.4% | 0 | 0.0% | 0 | 0.0% | 15 | 18.3% |
|  |  | 73.3% |  | 26.7% |  | 0.0% |  | 0.0% |  | 100.0% |  |
|  | nos | 18 | 14.9% | 6 | 20.7% | 0 | 0.0% | 0 | 0.0% | 24 | 15.7% |
|  |  | 75.0% |  | 25.0% |  | 0.0% |  | 0.0% |  | 100.0% |  |
|  | pen | 54 | 48.2% | 12 | 41.4% | 0 | 0.0% | 1 | 33.3% | 67 | 46.5% |
|  |  | 80.6% |  | 17.9% |  | 0.0% |  | 1.5% |  | 100.0% |  |
| Ternopil | Total | 71 | 4.9% | 22 | 2.9% | 1 | 1.6% | 1 | 4.3% | 95 | 4.2% |
|  |  | 74.7% |  | 23.2% |  | 1.1% |  | 1.0% |  | 100.0% |  |
|  | het | 70 | 98.6% | 22 | 100.0% | 1 | 100.0% | 1 | 100.0% | 94 | 98.9% |
|  |  | 74.5% |  | 23.4% |  | 1.1% |  | 1.0% |  | 100.0% |  |
|  | hrh | 18 | 25.4% | 12 | 54.5% | 0 | 0.0% | 1 | 100.0% | 31 | 32.6% |
|  |  | 58.1% |  | 38.7% |  | 0.0% |  | 3.2% |  | 100.0% |  |
|  | sti | 14 | 20.0% | 7 | 31.8% | 0 | 0.0% | 0 | 0.0% | 21 | 22.3% |
|  |  | 66.7% |  | 33.3% |  | 0.0% |  | 0.0% |  | 100.0% |  |
|  | idu | 2 | 2.8% | 19 | 86.4% | 0 | 0.0% | 1 | 100.0% | 22 | 23.2% |
|  |  | 9.1% |  | 86.4% |  | 0.0% |  | 4.5% |  | 100.0% |  |
|  | hcv | 26 | 37.7% | 19 | 86.4% | 0 | 0.0% | 0 | 0.0% | 45 | 48.4% |
|  |  | 57.8% |  | 42.2% |  | 0.0% |  | 0.0% |  | 100.0% |  |
|  | hbv | 17 | 24.6% | 14 | 63.6% | 0 | 0.0% | 1 | 100.0% | 32 | 34.4% |
|  |  | 53.1% |  | 43.8% |  | 0.0% |  | 3.1% |  | 100.0% |  |
|  | msm | 3 | 7.5% | 0 | 0.0% | 0 | 0.0% | 0 | 0.0% | 3 | 5.3% |
|  |  | 100.0% |  | 0.0% |  | 0.0% |  | 0.0% |  | 100.0% |  |
|  | nos | 3 | 4.3% | 0 | 0.0% | 0 | 0.0% | 0 | 0.0% | 3 | 3.2% |
|  |  | 100.0% |  | 0.0% |  | 0.0% |  | 0.0% |  | 100.0% |  |
|  | pen | 12 | 17.1% | 11 | 52.4% | 0 | 0.0% | 1 | 100.0% | 24 | 25.8% |
|  |  | 50.0% |  | 45.8% |  | 0.0% |  | 4.2% |  | 100.0% |  |
| Kharkiv | Total | 106 | 7.3% | 43 | 5.7% | 6 | 9.5% | 5 | 21.7% | 160 | 7.0% |
|  |  | 66.3% |  | 26.9% |  | 3.8% |  | 3.0% |  | 100.0% |  |
|  | het | 102 | 96.2% | 43 | 100.0% | 5 | 83.3% | 5 | 100.0% | 155 | 96.9% |
|  |  | 65.8% |  | 27.7% |  | 3.2% |  | 3.3% |  | 100.0% |  |
|  | hrh | 29 | 27.4% | 32 | 74.4% | 2 | 33.3% | 1 | 20.0% | 64 | 40.0% |
|  |  | 45.3% |  | 50.0% |  | 3.1% |  | 1.6% |  | 100.0% |  |
|  | sti | 23 | 21.7% | 15 | 35.7% | 3 | 60.0% | 1 | 20.0% | 42 | 26.6% |
|  |  | 54.8% |  | 35.7% |  | 7.1% |  | 2.4% |  | 100.0% |  |
|  | idu | 6 | 5.7% | 36 | 83.7% | 1 | 20.0% | 0 | 0.0% | 43 | 27.0% |
|  |  | 14.0% |  | 83.7% |  | 2.3% |  | 0.0% |  | 100.0% |  |
|  | hcv | 16 | 15.1% | 36 | 83.7% | 1 | 16.7% | 0 | 0.0% | 53 | 33.1% |
|  |  | 30.2% |  | 67.9% |  | 1.9% |  | 0.0% |  | 100.0% |  |
|  | hbv | 20 | 18.9% | 26 | 60.5% | 3 | 50.0% | 0 | 0.0% | 49 | 30.6% |
|  |  | 40.8% |  | 53.1% |  | 6.1% |  | 0.0% |  | 100.0% |  |
|  | msm | 8 | 22.2% | 1 | 2.9% | 4 | 66.7% | 1 | 33.3% | 14 | 17.7% |
|  |  | 57.1% |  | 7.1% |  | 28.6% |  | 7.2% |  | 100.0% |  |
|  | nos | 17 | 16.3% | 5 | 11.6% | 1 | 16.7% | 1 | 20.0% | 24 | 15.2% |
|  |  | 70.8% |  | 20.8% |  | 4.2% |  | 4.2% |  | 100.0% |  |
|  | pen | 43 | 43.0% | 22 | 55.0% | 2 | 66.7% | 3 | 60.0% | 70 | 47.3% |
|  |  | 61.4% |  | 31.4% |  | 2.9% |  | 4.3% |  | 100.0% |  |
| Chernivtsi | Total | 27 | 1.9% | 9 | 1.2% | 0 | 0.0% | 0 | 0.0% | 36 | 1.6% |
|  |  | 75.0% |  | 25.0% |  | 0.0% |  | 0.0% |  | 100.0% |  |
|  | het | 26 | 96.3% | 9 | 100.0% | 0 | 0.0% | 0 | 0.0% | 35 | 97.2% |
|  |  | 74.3% |  | 25.7% |  | 0.0% |  | 0.0% |  | 100.0% |  |
|  | hrh | 8 | 29.6% | 7 | 77.8% | 0 | 0.0% | 0 | 0.0% | 15 | 41.7% |
|  |  | 53.3% |  | 46.7% |  | 0.0% |  | 0.0% |  | 100.0% |  |
|  | sti | 5 | 19.2% | 5 | 62.5% | 0 | 0.0% | 0 | 0.0% | 10 | 29.4% |
|  |  | 50.0% |  | 50.0% |  | 0.0% |  | 0.0% |  | 100.0% |  |
|  | idu | 3 | 12.0% | 9 | 100.0% | 0 | 0.0% | 0 | 0.0% | 12 | 35.3% |
|  |  | 25.0% |  | 75.0% |  | 0.0% |  | 0.0% |  | 100.0% |  |
|  | hcv | 6 | 22.2% | 9 | 100.0% | 0 | 0.0% | 0 | 0.0% | 15 | 41.7% |
|  |  | 40.0% |  | 60.0% |  | 0.0% |  | 0.0% |  | 100.0% |  |
|  | hbv | 9 | 33.3% | 8 | 88.9% | 0 | 0.0% | 0 | 0.0% | 17 | 47.2% |
|  |  | 52.9% |  | 47.1% |  | 0.0% |  | 0.0% |  | 100.0% |  |
|  | msm | 2 | 15.4% | 0 | 0.0% | 0 | 0.0% | 0 | 0.0% | 2 | 10.0% |
|  |  | 100.0% |  | 0.0% |  | 0.0% |  | 0.0% |  | 100.0% |  |
|  | nos | 5 | 19.2% | 1 | 12.5% | 0 | 0.0% | 0 | 0.0% | 6 | 17.6% |
|  |  | 83.3% |  | 16.7% |  | 0.0% |  | 0.0% |  | 100.0% |  |
|  | pen | 10 | 40.0% | 6 | 66.7% | 0 | 0.0% | 0 | 0.0% | 16 | 47.1% |
|  |  | 62.5% |  | 37.5% |  | 0.0% |  | 0.0% |  | 100.0% |  |
| Total |  | 1443 | 100.0% | 756 | 100.0% | 63 | 100.0% | 23 | 100.0% | 2285 | 100.0% |
|  |  | 63.2% |  | 33.1% |  | 2.8% |  | 0.9% |  | 100.0% |  |
|  | het | 1414 | 98.0% | 740 | 97.9% | 43 | 68.3% | 22 | 95.7% | 2219 | 97.1% |
|  |  | 63.7% |  | 33.3% |  | 1.9% |  | 1.1% |  | 100.0% |  |
|  | hrh | 638 | 44.4% | 540 | 71.5% | 13 | 20.6% | 10 | 43.5% | 1201 | 52.7% |
|  |  | 53.1% |  | 45.0% |  | 1.1% |  | 0.8% |  | 100.0% |  |
|  | sti | 507 | 37.1% | 322 | 45.7% | 32 | 53.3% | 7 | 31.8% | 868 | 40.3% |
|  |  | 58.4% |  | 37.1% |  | 3.7% |  | 0.8% |  | 100.0% |  |
|  | idu | 356 | 24.9% | 641 | 85.0% | 7 | 11.3% | 11 | 47.8% | 1015 | 44.8% |
|  |  | 35.1% |  | 63.2% |  | 0.7% |  | 1.0% |  | 100.0% |  |
|  | hcv | 594 | 42.2% | 573 | 77.7% | 8 | 13.3% | 6 | 27.3% | 1181 | 53.1% |
|  |  | 50.3% |  | 48.5% |  | 0.7% |  | 0.5% |  | 100.0% |  |
|  | hbv | 541 | 38.5% | 439 | 59.6% | 29 | 48.3% | 5 | 22.7% | 1014 | 45.6% |
|  |  | 53.4% |  | 43.3% |  | 2.9% |  | 0.4% |  | 100.0% |  |
|  | msm | 46 | 7.8% | 34 | 6.1% | 49 | 83.1% | 1 | 9.1% | 130 | 10.6% |
|  |  | 35.4% |  | 26.2% |  | 37.7% |  | 0.7% |  | 100.0% |  |
|  | nos | 256 | 18.2% | 82 | 11.2% | 8 | 13.3% | 2 | 9.5% | 348 | 15.7% |
|  |  | 73.6% |  | 23.6% |  | 2.3% |  | 0.5% |  | 100.0% |  |
|  | pen | 783 | 58.6% | 517 | 74.2% | 24 | 46.2% | 14 | 66.7% | 1338 | 63.5% |
|  |  | 58.5% |  | 38.6% |  | 1.8% |  | 1.1% |  | 100.0% |  |

Risk factors (for definitions see Table 1): het – heterosexual exposure; hrh – high-risk heterosexual exposure; sti – sexually transmitted infections; idu – injecting drug use; hcv – exposure to HCV; hbv – exposure to HBV; msm – homosexual exposure; nos – nosocomial exposure; pen – skin penetration exposure.

Modes of transmission: HET – heterosexual; IDU – injecting drug use; MSM – homosexual; OTH – other.

# Table S5. Misclassification of modes of transmission by region.

|  | | Registry (verified records) | | Survey | | p-value | | Misclassification | Extrapolation coefficient |
| --- | --- | --- | --- | --- | --- | --- | --- | --- | --- |
|  |  | N | MoT % | N | MoT % | McNemar | Paired t-test |  |  |
| Dnipro | |  |  |  |  |  |  |  |  |
|  | HET | 796 | 64.8% | 416 | 33.8% | <0.001 | <0.001 | 91.3% | 52.3% |
|  | IDU | 410 | 33.4% | 801 | 65.2% | <0.001 | <0.001 | -48.8% | 195.4% |
|  | MSM | 10 | 0.8% | 8 | 0.7% | 0.804 | 0.617 | 25.0% | 80.0% |
|  | UNK | 13 | 1.1% | 4 | 0.3% | 0.035 | 0.020 | 225.0% | 30.8% |
| Kyiv |  |  |  |  |  |  |  |  |  |
|  | HET | 192 | 43.1% | 134 | 30.1% | <0.001 | <0.001 | 43.3% | 69.8% |
|  | IDU | 206 | 46.3% | 262 | 58.9% | <0.001 | <0.001 | -21.4% | 127.2% |
|  | MSM | 46 | 10.3% | 48 | 10.8% | 0.754 | 0.528 | -4.2% | 104.3% |
|  | UNK | 1 | 0.2% | 1 | 0.2% | 1.000 | 1.000 | 0.0% | 100.0% |
| Kropivnitsky | |  |  |  |  |  |  |  |  |
|  | HET | 124 | 78.0% | 56 | 35.2% | <0.001 | <0.001 | 121.4% | 45.2% |
|  | IDU | 35 | 22.0% | 102 | 64.2% | <0.001 | <0.001 | -65.7% | 291.4% |
|  | MSM | 0 | 0.0% | 1 | 0.6% | 1.000 | 0.319 | -100.0% | 0.6% |
|  | UNK | 0 | 0.0% | 0 | 0.0% | <0.001 | <0.001 | 0.0% | 0.0% |
| Lviv |  |  |  |  |  |  |  |  |  |
|  | HET | 120 | 77.9% | 66 | 42.9% | <0.001 | <0.001 | 81.8% | 55.0% |
|  | IDU | 31 | 20.1% | 75 | 48.7% | <0.001 | <0.001 | -58.7% | 241.9% |
|  | MSM | 0 | 0.0% | 13 | 8.4% | <0.001 | <0.001 | -100.0% | 8.4% |
|  | UNK | 3 | 1.9% | 0 | 0.0% | 0.250 | 0.083 | 0.0% | 0.0% |
| Ternopil | |  |  |  |  |  |  |  |  |
|  | HET | 71 | 74.7% | 44 | 46.3% | <0.001 | <0.001 | 61.4% | 62.0% |
|  | IDU | 22 | 23.2% | 48 | 50.5% | <0.001 | <0.001 | -54.2% | 218.2% |
|  | MSM | 1 | 1.1% | 3 | 3.2% | 0.625 | 0.320 | -66.7% | 300.0% |
|  | UNK | 1 | 1.1% | 0 | 0.0% | 1.000 | 0.320 | 0.0% | 0.0% |
| Kharkiv | |  |  |  |  |  |  |  |  |
|  | HET | 106 | 66.3% | 90 | 56.3% | 0.018 | 0.011 | 17.8% | 84.9% |
|  | IDU | 43 | 26.9% | 58 | 36.3% | 0.007 | 0.004 | -25.9% | 134.9% |
|  | MSM | 6 | 3.8% | 11 | 6.9% | 0.267 | 0.166 | -45.5% | 183.3% |
|  | UNK | 5 | 3.1% | 1 | 0.6% | 0.219 | 0.103 | 400.0% | 20.0% |
| Chernivtsi | |  |  |  |  |  |  |  |  |
|  | HET | 27 | 75.0% | 19 | 52.8% | 0.008 | 0.003 | 42.1% | 70.4% |
|  | IDU | 9 | 25.0% | 15 | 41.7% | 0.031 | 0.012 | -40.0% | 166.7% |
|  | MSM | 0 | 0.0% | 2 | 5.6% | 0.500 | 0.160 | -100.0% | 5.6% |
|  | UNK | 0 | 0.0% | 0 | 0.0% | <0.001 | <0.001 | 0.0% | 0.0% |
| Grand Total | |  |  |  |  |  |  |  |  |
|  | HET | 1436 | 63.0% | 825 | 36.2% | <0.001 | <0.001 | 74.1% | 57.5% |
|  | IDU | 756 | 33.2% | 1361 | 59.7% | <0.001 | <0.001 | -44.5% | 180.0% |
|  | MSM | 63 | 2.8% | 86 | 3.8% | 0.004 | 0.003 | -26.7% | 136.5% |
|  | UNK | 23 | 1.0% | 6 | 0.3% | 0.002 | 0.001 | 283.3% | 26.1% |

Modes of transmission: HET – heterosexual; IDU – injecting drug use; MSM – homosexual; OTH – other.

# Table S6. Trends in modes of transmission by region in the official reports.

|  |  | 2013 | 2014 | 2015 | 2013 | 2014 | 2015 | p-value for trend |
| --- | --- | --- | --- | --- | --- | --- | --- | --- |
|  |  | N | N | N | MoT % | MoT % | MoT % |  |
| Dnipro |  | 757 | 519 | 660 |  |  |  | 0.833 |
|  | HET | 488 | 334 | 422 | 64.5% | 64.4% | 63.9% | 0.838 |
|  | IDU | 259 | 178 | 231 | 34.2% | 34.3% | 35.0% | 0.760 |
|  | MSM | 2 | 2 | 4 | 0.3% | 0.4% | 0.6% | 0.319 |
|  | OTH | 8 | 5 | 3 | 1.1% | 1.0% | 0.5% | 0.217 |
| Kyiv |  | 317 | 295 | 267 |  |  |  | 0.334 |
|  | HET | 169 | 150 | 141 | 53.3% | 50.8% | 52.8% | 0.879 |
|  | IDU | 108 | 107 | 86 | 34.1% | 36.3% | 32.2% | 0.670 |
|  | MSM | 36 | 37 | 40 | 11.4% | 12.5% | 15.0% | 0.196 |
|  | OTH | 4 | 1 | 0 | 1.3% | 0.3% | 0.0% | 0.041 |
| Kropivnitsky |  | 87 | 108 | 90 |  |  |  | 0.960 |
|  | HET | 67 | 85 | 70 | 77.0% | 78.7% | 77.8% | 0.905 |
|  | IDU | 20 | 23 | 20 | 23.0% | 21.3% | 22.2% | 0.905 |
|  | MSM | 0 | 0 | 0 | 0.0% | 0.0% | 0.0% |  |
|  | OTH | 0 | 0 | 0 | 0.0% | 0.0% | 0.0% |  |
| Lviv |  | 71 | 136 | 114 |  |  |  | 0.009 |
|  | HET | 37 | 68 | 71 | 52.1% | 50.0% | 62.3% | 0.118 |
|  | IDU | 28 | 63 | 30 | 39.4% | 46.3% | 26.3% | 0.029 |
|  | MSM | 6 | 5 | 13 | 8.5% | 3.7% | 11.4% | 0.283 |
|  | OTH | 0 | 0 | 0 | 0.0% | 0.0% | 0.0% |  |
| Ternopil |  | 36 | 28 | 24 |  |  |  | 0.154 |
|  | HET | 26 | 19 | 18 | 72.2% | 67.9% | 75.0% | 0.865 |
|  | IDU | 10 | 9 | 4 | 27.8% | 32.1% | 16.7% | 0.396 |
|  | MSM | 0 | 0 | 0 | 0.0% | 0.0% | 0.0% |  |
|  | OTH | 0 | 0 | 2 | 0.0% | 0.0% | 8.3% | 0.047 |
| Kharkiv |  | 136 | 107 | 110 |  |  |  | 0.200 |
|  | HET | 64 | 57 | 72 | 47.1% | 53.3% | 65.5% | 0.004 |
|  | IDU | 65 | 47 | 34 | 47.8% | 43.9% | 30.9% | 0.009 |
|  | MSM | 3 | 3 | 4 | 2.2% | 2.8% | 3.6% | 0.503 |
|  | OTH | 4 | 0 | 0 | 2.9% | 0.0% | 0.0% | 0.025 |
| Chernivtsi |  | 17 | 16 | 18 |  |  |  | 0.910 |
|  | HET | 17 | 12 | 14 | 100.0% | 75.0% | 77.8% | 0.077 |
|  | IDU | 0 | 4 | 4 | 0.0% | 25.0% | 22.2% | 0.077 |
|  | MSM | 0 | 0 | 0 | 0.0% | 0.0% | 0.0% |  |
|  | OTH | 0 | 0 | 0 | 0.0% | 0.0% | 0.0% |  |
| Total |  | 1421 | 1209 | 1283 |  |  |  | 0.035 |
|  | HET | 868 | 725 | 808 | 61.1% | 60.0% | 63.0% | 0.329 |
|  | IDU | 490 | 431 | 409 | 34.5% | 35.6% | 31.9% | 0.166 |
|  | MSM | 47 | 47 | 61 | 3.3% | 3.9% | 4.8% | 0.055 |
|  | OTH | 16 | 6 | 5 | 1.1% | 0.5% | 0.4% | 0.019 |

Modes of transmission: HET – heterosexual; IDU – injecting drug use; MSM – homosexual; OTH – other.

# Table S7. Trends in modes of transmission by region in the verified registry.

|  |  | 2013 | 2014 | 2015 | 2013 | 2014 | 2015 | p-value for trend |
| --- | --- | --- | --- | --- | --- | --- | --- | --- |
|  |  | N | N | N | MoT % | MoT % | MoT % |  |
| Dnipro |  | 583 | 437 | 648 |  |  |  | 0.365 |
|  | HET | 383 | 284 | 415 | 65.7% | 65.0% | 64.0% | 0.544 |
|  | IDU | 192 | 141 | 224 | 32.9% | 32.3% | 34.6% | 0.534 |
|  | MSM | 5 | 6 | 2 | 0.9% | 1.4% | 0.3% | 0.255 |
|  | OTH | 3 | 6 | 7 | 0.5% | 1.4% | 1.1% | 0.323 |
| Kyiv |  | 318 | 299 | 275 |  |  |  | 0.704 |
|  | HET | 169 | 154 | 146 | 53.1% | 51.5% | 53.1% | 0.974 |
|  | IDU | 109 | 106 | 87 | 34.3% | 35.5% | 31.6% | 0.518 |
|  | MSM | 38 | 38 | 42 | 11.9% | 12.7% | 15.3% | 0.240 |
|  | OTH | 2 | 1 | 0 | 0.6% | 0.3% | 0.0% | 0.188 |
| Kropivnitsky |  | 87 | 107 | 90 |  |  |  | 0.790 |
|  | HET | 67 | 83 | 70 | 77.0% | 77.6% | 77.8% | 0.903 |
|  | IDU | 20 | 23 | 20 | 23.0% | 21.5% | 22.2% | 0.904 |
|  | MSM | 0 | 0 | 0 | 0.0% | 0.0% | 0.0% |  |
|  | OTH | 0 | 1 | 0 | 0.0% | 0.9% | 0.0% | 0.989 |
| Lviv |  | 64 | 108 | 99 |  |  |  | 0.637 |
|  | HET | 49 | 82 | 71 | 76.6% | 75.9% | 71.7% | 0.457 |
|  | IDU | 14 | 26 | 26 | 21.9% | 24.1% | 26.3% | 0.521 |
|  | MSM | 0 | 0 | 0 | 0.0% | 0.0% | 0.0% |  |
|  | OTH | 1 | 0 | 2 | 1.6% | 0.0% | 2.0% | 0.643 |
| Ternopil |  | 44 | 37 | 27 |  |  |  | 0.097 |
|  | HET | 34 | 29 | 19 | 77.3% | 78.4% | 70.4% | 0.556 |
|  | IDU | 10 | 4 | 8 | 22.7% | 10.8% | 29.6% | 0.662 |
|  | MSM | 0 | 3 | 0 | 0.0% | 8.1% | 0.0% | 0.729 |
|  | OTH | 0 | 1 | 0 | 0.0% | 2.7% | 0.0% | 0.843 |
| Kharkiv |  | 124 | 103 | 117 |  |  |  | 0.218 |
|  | HET | 54 | 52 | 71 | 43.5% | 50.5% | 60.7% | 0.008 |
|  | IDU | 64 | 45 | 41 | 51.6% | 43.7% | 35.0% | 0.010 |
|  | MSM | 2 | 3 | 3 | 1.6% | 2.9% | 2.6% | 0.620 |
|  | OTH | 4 | 3 | 2 | 3.2% | 2.9% | 1.7% | 0.464 |
| Chernivtsi |  | 19 | 19 | 22 |  |  |  | 0.411 |
|  | HET | 16 | 14 | 16 | 84.2% | 73.7% | 72.7% | 0.399 |
|  | IDU | 2 | 5 | 6 | 10.5% | 26.3% | 27.3% | 0.207 |
|  | MSM | 1 | 0 | 0 | 5.3% | 0.0% | 0.0% | 0.203 |
|  | OTH | 0 | 0 | 0 | 0.0% | 0.0% | 0.0% |  |
| Total |  | 1239 | 1110 | 1278 |  |  |  | 0.880 |
|  | HET | 772 | 698 | 808 | 62.3% | 62.9% | 63.2% | 0.635 |
|  | IDU | 411 | 350 | 412 | 33.2% | 31.5% | 32.2% | 0.621 |
|  | MSM | 46 | 50 | 47 | 3.7% | 4.5% | 3.7% | 0.956 |
|  | OTH | 10 | 12 | 11 | 0.8% | 1.1% | 0.9% | 0.892 |

Modes of transmission: HET – heterosexual; IDU – injecting drug use; MSM – homosexual; OTH – other.

# Table S8. Trends in modes of transmission by region in the survey.

|  |  | 2013 | 2014 | 2015 | 2013 | 2014 | 2015 | p-value for trend |
| --- | --- | --- | --- | --- | --- | --- | --- | --- |
|  |  | N | N | N | MoT % | MoT % | MoT % |  |
| Dnipro |  | 437 | 333 | 459 |  |  |  | 0.513 |
|  | HET | 151 | 100 | 165 | 34.6% | 30.0% | 35.9% | 0.644 |
|  | IDU | 282 | 227 | 292 | 64.5% | 68.2% | 63.6% | 0.761 |
|  | MSM | 3 | 3 | 2 | 0.7% | 0.9% | 0.4% | 0.635 |
|  | OTH | 1 | 3 | 0 | 0.2% | 0.9% | 0.0% | 0.530 |
| Kyiv |  | 158 | 135 | 152 |  |  |  | 0.470 |
|  | HET | 42 | 47 | 45 | 26.6% | 34.8% | 29.6% | 0.552 |
|  | IDU | 104 | 74 | 84 | 65.8% | 54.8% | 55.3% | 0.058 |
|  | MSM | 12 | 14 | 22 | 7.6% | 10.4% | 14.5% | 0.051 |
|  | OTH | 0 | 0 | 1 | 0.0% | 0.0% | 0.7% | 0.225 |
| Kropivnitsky |  | 49 | 59 | 51 |  |  |  | 0.950 |
|  | HET | 14 | 27 | 15 | 28.6% | 45.8% | 29.4% | 0.951 |
|  | IDU | 35 | 31 | 36 | 71.4% | 52.5% | 70.6% | 0.953 |
|  | MSM | 0 | 1 | 0 | 0.0% | 1.7% | 0.0% | 0.987 |
|  | OTH | 0 | 0 | 0 | 0.0% | 0.0% | 0.0% |  |
| Lviv |  | 39 | 60 | 55 |  |  |  | 0.083 |
|  | HET | 17 | 29 | 20 | 43.6% | 48.3% | 36.4% | 0.419 |
|  | IDU | 21 | 28 | 26 | 53.8% | 46.7% | 47.3% | 0.562 |
|  | MSM | 1 | 3 | 9 | 2.6% | 5.0% | 16.4% | 0.013 |
|  | OTH | 0 | 0 | 0 | 0.0% | 0.0% | 0.0% |  |
| Ternopil |  | 39 | 29 | 27 |  |  |  | 0.248 |
|  | HET | 16 | 13 | 15 | 41.0% | 44.8% | 55.6% | 0.258 |
|  | IDU | 22 | 14 | 12 | 56.4% | 48.3% | 44.4% | 0.329 |
|  | MSM | 1 | 2 | 0 | 2.6% | 6.9% | 0.0% | 0.660 |
|  | OTH | 0 | 0 | 0 | 0.0% | 0.0% | 0.0% |  |
| Kharkiv |  | 35 | 52 | 73 |  |  |  | 0.725 |
|  | HET | 17 | 32 | 41 | 48.6% | 61.5% | 56.2% | 0.596 |
|  | IDU | 16 | 19 | 23 | 45.7% | 36.5% | 31.5% | 0.158 |
|  | MSM | 2 | 1 | 8 | 5.7% | 1.9% | 11.0% | 0.180 |
|  | OTH | 0 | 0 | 1 | 0.0% | 0.0% | 1.4% | 0.332 |
| Chernivtsi |  | 9 | 13 | 14 |  |  |  | 0.412 |
|  | HET | 5 | 7 | 7 | 55.6% | 53.8% | 50.0% | 0.789 |
|  | IDU | 4 | 6 | 5 | 44.4% | 46.2% | 35.7% | 0.646 |
|  | MSM | 0 | 0 | 2 | 0.0% | 0.0% | 14.3% | 0.116 |
|  | OTH | 0 | 0 | 0 | 0.0% | 0.0% | 0.0% |  |
| Total |  | 766 | 681 | 831 |  |  |  | 0.020 |
|  | HET | 262 | 255 | 308 | 34.2% | 37.4% | 37.1% | 0.242 |
|  | IDU | 484 | 399 | 478 | 63.2% | 58.6% | 57.5% | 0.022 |
|  | MSM | 19 | 24 | 43 | 2.5% | 3.5% | 5.2% | 0.005 |
|  | OTH | 1 | 3 | 2 | 0.1% | 0.4% | 0.2% | 0.686 |

Modes of transmission: HET – heterosexual; IDU – injecting drug use; MSM – homosexual; OTH – other.

# Table S9. Trends in risk factors by region in the survey.

|  |  | 2013 | 2014 | 2015 | 2013 | 2014 | 2015 | p-value for trend |
| --- | --- | --- | --- | --- | --- | --- | --- | --- |
|  |  | N | N | N | % | % | % |  |
| Dnipro |  | 438 | 334 | 460 |  |  |  |  |
|  | het | 434 | 321 | 446 | 99.1% | 96.1% | 97.0% | 0.060 |
|  | hrh | 246 | 185 | 254 | 56.2% | 56.2% | 55.5% | 0.940 |
|  | sti | 174 | 142 | 222 | 45.2% | 44.4% | 52.5% | 0.027 |
|  | idu | 203 | 172 | 214 | 46.7% | 51.8% | 47.1% | 0.904 |
|  | hcv | 229 | 200 | 239 | 52.5% | 60.2% | 52.0% | 0.971 |
|  | hbv | 202 | 154 | 203 | 46.3% | 46.4% | 44.1% | 0.657 |
|  | msm | 9 | 10 | 11 | 4.0% | 5.3% | 4.8% | 0.695 |
|  | nos | 60 | 66 | 72 | 14.1% | 20.3% | 16.3% | 0.535 |
|  | pen | 303 | 207 | 289 | 73.0% | 68.3% | 66.7% | 0.079 |
| Kyiv |  | 158 | 135 | 153 |  |  |  |  |
|  | het | 154 | 132 | 139 | 97.5% | 97.8% | 90.8% | 0.006 |
|  | hrh | 100 | 86 | 76 | 63.3% | 63.7% | 49.7% | 0.018 |
|  | sti | 70 | 57 | 71 | 45.5% | 43.2% | 46.7% | 0.786 |
|  | idu | 88 | 67 | 68 | 55.7% | 49.6% | 44.4% | 0.054 |
|  | hcv | 98 | 60 | 77 | 62.4% | 58.8% | 53.8% | 0.152 |
|  | hbv | 85 | 56 | 71 | 54.1% | 54.9% | 50.0% | 0.522 |
|  | msm | 13 | 18 | 29 | 14.8% | 24.7% | 30.2% | 0.014 |
|  | nos | 11 | 17 | 30 | 7.3% | 13.0% | 20.1% | 0.001 |
|  | pen | 95 | 90 | 97 | 72.5% | 72.6% | 72.9% | 0.860 |
| Kropivnitsky | | 49 | 59 | 51 |  |  |  |  |
|  | het | 48 | 59 | 50 | 98.0% | 100.0% | 98.0% | 0.991 |
|  | hrh | 27 | 35 | 29 | 55.1% | 59.3% | 56.9% | 0.773 |
|  | sti | 7 | 12 | 14 | 14.3% | 20.7% | 29.2% | 0.075 |
|  | idu | 24 | 25 | 26 | 49.0% | 42.4% | 52.0% | 0.760 |
|  | hcv | 30 | 31 | 34 | 63.8% | 52.5% | 66.7% | 0.652 |
|  | hbv | 22 | 25 | 34 | 46.8% | 42.4% | 66.7% | 0.034 |
|  | msm | 0 | 2 | 4 | 0.0% | 7.1% | 13.8% | 0.047 |
|  | nos | 3 | 17 | 15 | 6.4% | 28.8% | 30.0% | 0.005 |
|  | pen | 19 | 27 | 34 | 45.2% | 47.4% | 69.4% | 0.013 |
| Lviv |  | 39 | 63 | 55 |  |  |  |  |
|  | het | 39 | 61 | 52 | 100.0% | 96.8% | 94.5% | 0.040 |
|  | hrh | 15 | 17 | 21 | 39.5% | 27.0% | 38.2% | 0.971 |
|  | sti | 3 | 12 | 11 | 7.9% | 19.7% | 23.4% | 0.059 |
|  | idu | 16 | 17 | 18 | 41.0% | 27.0% | 34.0% | 0.550 |
|  | hcv | 20 | 27 | 23 | 54.1% | 44.3% | 44.2% | 0.481 |
|  | hbv | 16 | 27 | 21 | 43.2% | 44.3% | 40.4% | 0.721 |
|  | msm | 1 | 4 | 10 | 5.3% | 13.8% | 29.4% | 0.037 |
|  | nos | 2 | 14 | 8 | 5.4% | 22.2% | 15.1% | 0.258 |
|  | pen | 14 | 24 | 29 | 37.8% | 42.9% | 56.9% | 0.066 |
| Ternopil |  | 39 | 29 | 27 |  |  |  |  |
|  | het | 39 | 28 | 27 | 100.0% | 96.6% | 100.0% | 0.878 |
|  | hrh | 15 | 5 | 11 | 38.5% | 17.2% | 40.7% | 0.982 |
|  | sti | 7 | 5 | 9 | 18.4% | 17.2% | 33.3% | 0.182 |
|  | idu | 10 | 6 | 6 | 25.6% | 20.7% | 22.2% | 0.720 |
|  | hcv | 21 | 13 | 11 | 55.3% | 46.4% | 40.7% | 0.243 |
|  | hbv | 12 | 10 | 10 | 31.6% | 35.7% | 37.0% | 0.640 |
|  | msm | 1 | 2 | 0 | 4.8% | 11.1% | 0.0% | 0.549 |
|  | nos | 1 | 1 | 1 | 2.7% | 3.4% | 3.7% | 0.819 |
|  | pen | 10 | 5 | 9 | 26.3% | 17.2% | 34.6% | 0.546 |
| Kharkiv |  | 35 | 52 | 73 |  |  |  |  |
|  | het | 34 | 52 | 69 | 97.1% | 100.0% | 94.5% | 0.300 |
|  | hrh | 19 | 23 | 22 | 54.3% | 44.2% | 30.1% | 0.012 |
|  | sti | 12 | 16 | 14 | 34.3% | 32.0% | 19.2% | 0.066 |
|  | idu | 12 | 13 | 18 | 34.3% | 25.5% | 24.7% | 0.335 |
|  | hcv | 15 | 17 | 21 | 42.9% | 32.7% | 28.8% | 0.156 |
|  | hbv | 12 | 15 | 22 | 34.3% | 28.8% | 30.1% | 0.710 |
|  | msm | 2 | 2 | 10 | 9.5% | 10.0% | 26.3% | 0.085 |
|  | nos | 1 | 9 | 14 | 2.9% | 17.3% | 19.4% | 0.033 |
|  | pen | 16 | 24 | 30 | 47.1% | 50.0% | 45.5% | 0.814 |
| Chernivtsi |  | 9 | 13 | 14 |  |  |  |  |
|  | het | 9 | 13 | 13 | 100.0% | 100.0% | 92.9% | 0.268 |
|  | hrh | 3 | 7 | 5 | 33.3% | 53.8% | 35.7% | 0.920 |
|  | sti | 3 | 5 | 2 | 37.5% | 41.7% | 14.3% | 0.241 |
|  | idu | 3 | 4 | 5 | 33.3% | 33.3% | 38.5% | 0.794 |
|  | hcv | 4 | 6 | 5 | 44.4% | 46.2% | 35.7% | 0.780 |
|  | hbv | 4 | 6 | 7 | 44.4% | 46.2% | 50.0% | 0.634 |
|  | msm | 0 | 0 | 2 | 0.0% | 0.0% | 20.0% | 0.172 |
|  | nos | 2 | 0 | 4 | 25.0% | 0.0% | 30.8% | 0.475 |
|  | pen | 3 | 7 | 6 | 42.9% | 53.8% | 42.9% | 1.000 |
| Total |  | 767 | 685 | 833 |  |  |  |  |
|  | het | 757 | 666 | 796 | 98.7% | 97.2% | 95.6% | <0.001 |
|  | hrh | 425 | 358 | 418 | 55.5% | 52.6% | 50.3% | 0.057 |
|  | sti | 276 | 249 | 343 | 39.0% | 37.6% | 43.8% | 0.042 |
|  | idu | 356 | 304 | 355 | 46.6% | 44.6% | 43.1% | 0.172 |
|  | hcv | 417 | 354 | 410 | 54.9% | 54.7% | 50.0% | 0.092 |
|  | hbv | 353 | 293 | 368 | 46.5% | 45.3% | 44.9% | 0.692 |
|  | msm | 26 | 38 | 66 | 6.4% | 10.5% | 14.5% | <0.001 |
|  | nos | 80 | 124 | 144 | 10.8% | 18.5% | 17.8% | <0.001 |
|  | pen | 460 | 384 | 494 | 65.3% | 61.0% | 64.0% | 0.819 |

Risk factors (for definitions see Table 1): het – heterosexual exposure; hrh – high-risk heterosexual exposure; sti – sexually transmitted infections; idu – injecting drug use; hcv – exposure to HCV; hbv – exposure to HBV; msm – homosexual exposure; nos – nosocomial exposure; pen – skin penetration exposure.
