# Supplementary material for: Improved ascertainment of modes of HIV transmission in Ukraine indicates importance of drug injecting and homosexual risk
Source: BMC Public Health. 2020 Aug 26;20:1288. doi: 10.1186/s12889-020-09373-2 (PMC7449084; doi:10.1186/s12889-020-09373-2)
Supplement: Supplementary file 1 — Additional file 1: Supplementary file 1. Participant questionnaire. This is the original version of the study questionnaire in Ukrainian with machine-translated English version. [file 12889_2020_9373_MOESM1_ESM.pdf]

ASSESSMENT OF WAYS OF HIV TRANSMISSION IN UKRAINE

QUESTIONNAIRE OF THE RESEARCH PARTICIPANT

Dear research participant!

This study aims to assess the ways of HIV infection among patients in Ukraine who have been diagnosed with HIV. The results will be used to plan more effective measures to prevent and treat HIV in Ukraine. You will be one of more than 2,300 study participants. All information received from participants during the study will remain completely confidential. The results of the study will be published in a generalized form solely for scientific and practical purposes and will not contain any personal data.

This survey will take approximately 30 minutes of your time.

To be filled in by the interviewer:

**Site ID and serial № from the Log of enrollment**	*Site ID* *№ Log*
**Interview start date and time:**	*numeric* *month* 2 0 1 _ *year* _ _ : _ _ *time (YY: XX)*
**Date of birth of the respondent:** (*day / month / year*)	*numeric* *month* 1 9 _ _ *year*
**Sex at birth:**	1. Male 2.Female

To be filled in by the respondent:

COMMENT 1 : During this interview, you will be asked about certain events or circumstances you may have been through, such as medical procedures, drug use, travel, and sex. When answering questions, try to give the most accurate and complete answers.

Month	*The year you found out*

1. **Please try to remember the month and year you learned that you had HIV.**

	2 0 _ _
Month	*Year of registration*

2. **Please indicate the month and year when you registered with the AIDS Center or Trust.**

3. **Do you have sex with men / women at this time?**

□ Only with men ……………………… .1
□ Only with women ……………………… ... 2
□ Both with men and with women 3 .3
□ *I don't have sex ……………………… 4*
□ *I refuse to answer ……….. 998*

COMMENT 2 : All of the following questions cover the period of 10 years before you learned you had HIV .

The following questions are about your sex life. Sexual intercourse means vaginal, anal or oral sex with a man or woman.

4. **How many women have you had sex with in 10 years before you found out you had HIV?**
If you do not remember the exact number of partners, indicate the average. For example, if you had 3-5 partners, specify 4.

_______ woman / women
□ *I refuse to answer ………….. .999*

5. **How many men have you had sex with in 10 years before you found out you had HIV?**
If you do not remember the exact number of partners, indicate the average. For example, if you had 3-5 partners, specify 4.

_______ husband / men
□ *I refuse to answer ………….. .999*

Note: *If the answers to questions 4* ***and*** *5 are "None" and "None" (* ***0 partners*** *), please go to* ***question 17.***

6. In the 10 years before you learned you had HIV, what types of sexual contact did you have? *(Check all that apply)*

□ Vaginal 1
□ Anal 2
□ Oral 3

7. During the 10 years before you learned that you had HIV, did you have sex with a person of the same sex as you?

□ Yes 1
□ No 2
□ *Unknown* 998
□ *I refuse to answer* 999

8. In the 10 years before you found out you had HIV, how often did you use a condom during sexual intercourse?

□ Always (100%) 1
□ In most cases (~ 75%) 2
□ In half of the cases (~ 50%) 3
□ Sometimes (~ 25%) 4
□ Rarely (<10%) 5
□ Never 6
□ I *refuse to answer* 999

9. During the 10 years before you learned that you had HIV, did you have (homo- or hetero-) sexual contact with an injecting drug user?

□ Yes 1
□ No 2
□ *Unknown* 998
□ *I refuse to answer* 999

10. During the 10 years before you learned that you had HIV, did you have sexual intercourse while under the influence of alcohol or drugs?

□ Yes 1
□ No 2
□ *I refuse to answer* 999

11. Have you had sexual intercourse with a man who had a bisexual orientation?

□ Yes 1
□ No 2
□ *Unknown* 998
□ *I refuse to answer* 999

12. During the 10 years before you learned that you had HIV, did you have sexual intercourse with a person who is known to be HIV-positive or AIDS?

□ Yes 1
□ No 2
□ *Unknown* 998
□ *I refuse to answer* 999

Note: *If the answer is* ***NO*** *, please go to* ***question 15.***

13. If YES, were they homosexual or heterosexual?

□ Homosexual 1
□ Heterosexual 2
□ Homo- and heterosexual 3
□ *I refuse to answer* 999

14. Was this person known to have one of the following conditions? *(Please mark everything that was characteristic of this person / persons)*

□ Person with hemophilia / coagulation disorders 1
□ Person who has had a blood transfusion / blood components 2
□ Person who has had an organ transplant / transplant 3
□ A person who has used psychoactive substances (drugs) 4
□ *None of the above* 5
□ *I refuse to answer* 999

15. During this period (***10 years before you learned you had HIV*** **), did you have sexual contact with a commercial sex worker (ie a person who provides sexual services in exchange for drugs or money)?**

□ Yes 1
□ No 2
□ *Unknown* 998
□ *I refuse to answer* 999

16. In the 10 years before you found out you had HIV, did you have to receive money or drugs in exchange for sex?

□ Yes 1

□ No 2
□ *Unknown* 998
□ *I refuse to answer* 999

COMMENT 3: The following questions concern non-prescription drugs or narcotics and where they can be obtained.

17. Did you have 10 years of experience before you found out you had HIV to visit places where you could take illegal injecting drugs with other people? Even if you didn't use drugs there.

□ Yes 1
□ No 2
□ *Unknown* 998
□ *I refuse to answer* 999

18. Have you ever used any injectable drugs?

□ Yes 1
□ No 2
□ *I refuse to answer* 999

Note: *If* ***NO*** *, please go to* ***question 24.***

Age, years	

19. At what age did you start injecting drugs?

□ *I don't remember* ………………… ..999

20. How long has it been since your first injecting drug trial before you were diagnosed with HIV?

□ Less than a year 1
□ 1-3 years 2
□ 4-6 years 3
□ Over 6 years 4
□ *I don't remember 998*
□ *I refuse to answer 999*

21. In the <u>last 6 months</u> , how often have you used INJECTION drugs?

□ Daily 1
□ Several times a week 2
□ Once a week 3
□ Several times a month or less 4
□ *Did not use at all 5*
□ *I refuse to answer 999*

22. During the 10 years before you learned that you had HIV, that you had HIV, how often did you inject drugs?

□ Daily 1
□ Several times a week 2
□ Once a week 3
□ Several times a month or less 4
□ *Did not use at all 5*
□ *I refuse to answer 999*

Note: *If the answer is* **"Did not use at all"** *, please go to* ***question 24.***

23. Have you had to inject the following drugs by injection for 10 years before you found out you had HIV?

23.1. OPIATES (*Please tick all that* **apply)**

□ Desomorphine ("crocodile", "electroshock") 1
□ Tramadol / tramal 2
□ Heroin 3
□ Opium extract in liquid form ("shirka", "black") 4
□ Street methadone 5
□ Street buprenorphine 6
□ Morphine 7
□ *Other opiates, which are* : ________________________? 8
□ *Did not use opiates* 9

23.2. STIMULATORS (*Please tick all that match* **)**

□ Cocaine 1
□ Amphetamine ("hair dryer") (in powder form) 2
□ Methamphetamine powder (crystalline) 3
□ Methamphetamine in the form of a solution ("screw", "pervintin") - *drugs that are made with the addition of iodine and red phosphorus from drugs that contain ephedrine or pseudoephedrine* 4
□ Metcatinone ("Jeff") - *drugs that are made with the addition of manganese from drugs that contain ephedrine* 5
□ Katinon ("bovtushka" "Mulka") - *drugs that are made with the addition of manganese-based drug phenylpropanolamine (PPA)* 6
□ "Bath salt" 7
□ *Other stimulants, which are* : ________________________? 8
□ *Did not use stimulants* 9

23.3. PHARMACY OR MIXED (*Please tick all that apply* **)**

□ Pharmacy drugs (trapicamide, rinazoline, calypsol, ketamine) - *drugs purchased at the pharmacy and do not require special preparation for use for non-therapeutic purposes* 1
□ Pharmacy sedatives ("sonnate" (zopiclone), diphenhydramine) 2
□ Several substances in one syringe (opiates and barbiturates - diphenhydramine, tranquilizers and hypnotics) 3
□ *Other injecting drugs, which are* : ____________________? 4
□ *Not used* 5

24. During the 10 years before you learned that you had HIV, did you (or did you inject) yourself with any substances with a non-sterile or second-hand syringe?

□ Yes 1
□ No 2
□ *Unknown* 998
□ *I refuse to answer* 999

25. Within 10 years until you learned that you had HIV, were there any cases in your life when….

25.1. … .Do you get deep skin damage with needles or knives (***eg tattoos, scarring, religious rites*** **)?**

□ Yes 1
□ No 2
□ *Unknown* 998
□ *I refuse to answer* 999

25.2. … .Have you come across another person's blood on your damaged skin and / or mucous membranes?

□ Yes 1
□ No 2
□ *Unknown* 998
□ *I refuse to answer* 999

26. The following are questions about medical procedures you have had <u>for 10 years before</u> you learned you had HIV.

26.1. Have you had a blood transfusion or blood products such as coagulation factors? For example, due to hemophilia or other blood clotting disorders.

- *Coagulation factors - substances in blood plasma and platelets that provide blood clotting.*

□ Yes 1
□ No 2
□ *Unknown* 998
□ *I refuse to answer* 999

If YES, in what year / years? ____

□ *I don't remember the year / years* .... .999

26.2. Have you had an organ transplant / transplant? *For example, kidney, heart or lungs, cornea or bone graft.*

□ Yes 1
□ No 2
□ *Unknown* 998

□ *I refuse to answer* 999

If YES, in what year / years? ____

□ *I don't remember the year / years* .... .999

26.3. NOTE: Questions for women only!
Have you had an artificial insemination procedure while trying to get pregnant?

□ Yes 1
□ No 2
□ *Unknown* 998
□ *I refuse to answer* 999

If YES, in what year / years? ____

□ *I don't remember the year / years* .... .999

27. By the time you found out you had HIV, did you have such diseases?

*Please note everything that corresponds and the years when you were ill **.** Write 999 in the "Year / years" field, if you do not remember when it was.*		**Year / years when ill**
□ Hepatitis B	1	____________
□ Hepatitis C	2	____________
□ Hepatitis, what exactly - unknown	3	____________
□ Gonorrhea (including mouth or throat)	4	____________
□ Rectal gonorrhea (rectal gonorrhea) .... (.	5	____________
□ Syphilis	6	____________
□ Genital (genital) herpes (.	7	____________
□ proctitis (inflammation of the lining of the rectum)	8	____________
□ Any other sexually transmitted disease	9	____________
□ *Did not suffer from such diseases*	10	
□ *I refuse to answer*	999	

28. Have you been vaccinated against hepatitis B in the last 5 years?

□ Yes 1
□ No 2
□ *Unknown* 998
□ *I refuse to answer* 999

29. Have you worked in healthcare facilities or clinical laboratories?

□ Yes 1
□ No 2
□ *I refuse to answer* 999

30. Have you ever been in a pre-trial detention center, a penal colony or a pre-trial detention center (CPU)?

□ Yes 1
□ No 2
□ *I refuse to answer* 999

31. When do you think you could have been infected with HIV?

If less than a year, how many MONTHS ago did it happen?

If more than 12 months, how many YEARS ago did it happen?

32. How do you think you became infected with HIV?

□ Sexually, through *heterosexual* contact with an HIV-infected *partner who has NOT used injecting drugs* 1
□ Sexually, during *homosexual* contact with an HIV-infected *partner who has NOT used injecting drugs* 2
□ Sexually, through *heterosexual* contact with an HIV-infected *injecting drug partner* 3

- □ Sexually, through *homosexual* contact with an HIV-infected *injecting drug partner* 4
- □ Through the use of shared syringes / needles when injecting drugs 5
- □ Through the use of non-sterile medical instruments in a hospital or clinic 6
- □ During a blood transfusion (to you) that has been infected with HIV 7
- □ During an organ / tissue transplant from an HIV-infected donor 8
- □ Due to the use of non-sterile instruments when tattooing or piercing 9
- □ As a result of an accident at the workplace (during laboratory work, surgery, blood sampling, medical manipulations, etc.) 10
- □ *Other, what exactly* : ____? 11

COMMENT 5 : Thank you for taking the time to participate in this study. The information will help us a lot to learn more about HIV infection and ways of HIV infection in Ukraine.

		:		
(YY: XX)				

Interview end time :

1

АНКЕТА УЧАСНИКА ДОСЛІДЖЕННЯ

Шановний учаснику дослідження!

Дане дослідження має на меті оцінити шляхи інфікування ВІЛ серед пацієнтів в Україні, в яких було діагностовано ВІЛ-інфекцію. Результати буде використано для планування більш ефективних заходів профілактики та лікування ВІЛ-інфекції в Україні. Ви будете одним із більш ніж 2300 учасників дослідження. Вся інформація, отримана від учасників впродовж дослідження, буде залишатися повністю конфіденційною. Результати дослідження буде опубліковано в узагальненій формі виключно з науково-практичною метою і не будуть містити жодних персональних даних.

Дане опитування займе приблизно 30 хвилин Вашого часу.

Місце для штрих-коду

Заповнюється інтерв'юером:

**Код сайту та порядковий № з Журналу набору учасників**	[ ] ***Код сайту*** [ ][ ][ ] ***№ ЖНУ***
**Дата та час початку інтерв'ю:**	[ ][ ] ***число*** [ ][ ] ***місяць*** **2 0 1 6** ***рік*** [ ][ ] **:** [ ][ ] ***час (ГГ:ХХ)***
**Дата народження респондента:** (*день/місяць/рік*)	[ ][ ] ***число*** [ ][ ] ***місяць*** **1 9** [ ][ ] ***рік***
**Стать респондента:**	1. Чоловіча 2. Жіноча

Заповнюється респондентом:

КОМЕНТАР 1: Під час цього інтерв'ю Вам будуть задаватись запитання про певні події або обставини, через які Ви могли пройти — наприклад, такі як медичні процедури, споживання наркотиків, поїздки та сексуальне життя. Відповідаючи на запитання, намагайтеся давати найбільш точні та повні відповіді.

[ ][ ]	[ ][ ][ ][ ]
Місяць	***Рік, коли дізналися***

1. Спробуйте, будь ласка, згадати місяць і рік, коли Ви дізнались, що у Вас ВІЛ-інфекція.

			2	0		
Місяць			***Рік реєстрації***			

2. **Вкажіть, будь ласка, місяць і рік, коли Ви стали на облік у СНІД центр чи кабінет Довіри.**

3. **Чи маєте Ви статеві стосунки з чоловіками / жінками на даний період часу?**

□ Тільки з чоловіками……………………….1
□ Тільки з жінками…………………………...2
□ І з чоловіками, і з жінками…………….3
□ *Не займаюсь сексом………………………4*
□ *Відмовляюсь відповідати………..998*

КОМЕНТАР 2: Всі наступні запитання стосуються періоду <u>10 років до моменту, коли Ви дізнались, що у Вас ВІЛ</u>.

Наступні запитання стосуються Вашого статевого життя. <u>Під статевим контактом мається на увазі вагінальний, анальний або оральний секс з чоловіком або жінкою.</u>

4. **Зі скількома жінками Ви мали статеві контакти протягом 10 років до моменту, коли Ви дізнались, що у Вас ВІЛ?**
Якщо Ви не пам'ятаєте точну кількість партнерів, зазначте у середньому. Наприклад, якщо у Вас було 3-5 партнерів, зазначте 4.

_______ жінкою / жінками
□ *Відмовляюсь відповідати……………999*

5. **Зі скількома чоловіками Ви мали статевий зв'язок протягом 10 років до моменту, коли Ви дізнались, що у Вас ВІЛ?**
Якщо Ви не пам'ятаєте точну кількість партнерів, зазначте у середньому. Наприклад, якщо у Вас було 3-5 партнерів, зазначте 4.

_______ чоловіком / чоловіками
□ *Відмовляюсь відповідати……………999*

> ***Примітка:*** *Якщо відповідь на запитання 4* ***та*** *5 «З жодною» та «З жодним» (**0 партнерів**), перейдіть, будь ласка, до* ***запитання 17.***

6. **Протягом 10 років до моменту, коли Ви дізнались, що у Вас ВІЛ, які види сексуальних контактів Ви практикували?** *(Відмітьте всі відповідні варіанти)*

□ Вагінальні 1
□ Анальні 2
□ Оральні 3

7. **Протягом 10 років до моменту, коли Ви дізнались, що у Вас ВІЛ, чи вступали Ви у сексуальні контакти з особою тієї ж самої статі, що й Ви?**

□
Так

1
□
Ні

2
□
Невідомо

998
□ *Відмовляюся відповідати*

999

8. **Протягом 10 років до моменту, коли Ви дізнались, що у Вас ВІЛ, як часто Ви використовували презерватив під час сексуальних контактів?**

□ Завжди (100%)	1
□ У більшості випадків (~75%)	2
□ У половині випадків (~50%)	3
□ Іноді (~25%)	4
□ Рідко (< 10%)	5
□ Ніколи	6
□ *Відмовляюся відповідати* 999	

9. **Протягом 10 років до моменту, коли Ви дізнались, що у Вас ВІЛ, чи вступали Ви у (гомо- або гетеро-) сексуальні контакти з особою, що вживає наркотики ін'єкційним шляхом?**

□
Так

1
□
Ні

2
□
Невідомо

998

□ *Відмовляюся відповідати*

999

10. **Протягом 10 років до моменту, коли Ви дізнались, що у Вас ВІЛ, чи вступали Ви у сексуальні контакти, перебуваючи під впливом алкоголю або наркотиків?**

□ Так

1

□ Ні

2

□ *Відмовляюся відповідати*

999

11. **Чи вступали Ви у сексуальні контакти з чоловіком, який мав бісексуальну орієнтацію?**

□ Так

1

□ Ні

2

□ *Невідомо*

998

□ *Відмовляюся відповідати*

999

12. **Протягом 10 років до моменту, коли Ви дізнались, що у Вас ВІЛ, чи вступали Ви у сексуальні контакти з особою, про яку відомо, що вона ВІЛ-інфікована або хвора на СНІД?**

□ Так

1

□ Ні

2
□
Невідомо

998
□ *Відмовляюся відповідати*

999

Примітка: *Якщо відповідь **НІ**, перейдіть, будь ласка, до **запитання 15.***

13. Якщо ТАК, це були гомо- чи гетеросексуальні контакти?

□
Гомосексуальні

1
□
Гетеросексуальні

2
□ Гомо- та гетеросексуальні

3
□ *Відмовляюся відповідати*

999

14. Чи було відомо про цю особу, що вона мала один із нижче перелічених станів? *(Будь ласка, відмітьте все, що було характерно для цієї особи/осіб)*

□ Особа з гемофілією/порушеннями згортання крові

1
□ Особа, якій робили переливання крові/компонентів крові

2
□ Особа, якій робили пересадку/трансплантацію органів

3
□ Особа, яка вживала психоактивні речовини (наркотики)

4
□ *Нічого з переліченого вище*

5
□ *Відмовляюся відповідати*

999

15. **Протягом цього періоду (*протягом 10 років до моменту, коли Ви дізнались, що у Вас ВІЛ*), чи вступали Ви у сексуальні контакти з робітником комерційного сексу (тобто людиною, що надає сексуальні послуги в обмін на наркотики або гроші)?**

□ Так

1
□ Ні

2
□ *Невідомо*

998
□ *Відмовляюся відповідати*

999

16. **Протягом 10 років до моменту, коли Ви дізнались, що у Вас ВІЛ, чи доводилося Вам отримувати гроші або наркотики в обмін на секс?**

□ Так

1
□ Ні

2
□ *Невідомо*

998
□ *Відмовляюся відповідати*

999

КОМЕНТАР 3: Наступні запитання стосуються наркотичних речовин або наркотичних препаратів, що були відпущені НЕ за рецептом лікаря, а також місць, де їх можна отримати.

17. **Чи мали Ви досвід протягом 10 років до того моменту, коли Ви дізналися, що у Вас ВІЛ, відвідувати місця, де можна прийняти нелегальні ІН'ЄКЦІЙНІ наркотики разом з іншими людьми? Навіть, якщо Ви не вживали там наркотики.**

□ Так 1

□ Ні 2

□ *Невідомо* 998

□ *Відмовляюся відповідати* 999

18. **Чи вживали Ви коли-небудь будь-які наркотики ІН'ЄКЦІЙНИМ шляхом?**

□ Так 1

□ Ні 2

□ *Відмовляюся відповідати* 999

Примітка: *Якщо відповідь **НІ**, перейдіть, будь ласка, до **запитання 24.***

Вік, років	

19. У якому віці Ви почали вживати наркотики ІН'ЄКЦІЙНО?

□ *Не пам'ятаю*…………………..999

20. Скільки часу пройшло з моменту Вашої першої проби ін'єкційного наркотику до того, як у Вас виявили ВІЛ?

□ Менше року 1

□ 1-3 роки 2

□ 4-6 років 3

□ Понад 6 років 4

□ *Не пам'ятаю 998*

□ *Відмовляюся відповідати 999*

21. За <u>останні 6 місяців</u>, як часто Ви вживали ІН'ЄКЦІЙНІ наркотики?

□ Щодня 1

□ Кілька разів на тиждень 2

□ Раз на тиждень 3

□ Кілька разів на місяць або рідше

4
□ *Не вживав/ла взагалі*

5
□ *Відмовляюся відповідати*

999

22. Протягом 10 років до того моменту, коли Ви дізналися, що у Вас ВІЛ, що у Вас ВІЛ-інфекція, як часто Ви вживали ІН'ЄКЦІЙНІ наркотики?

□ Щодня

1
□ Кілька разів на тиждень

2
□ Раз на тиждень

3
□ Кілька разів на місяць або рідше

4
□ *Не вживав/ла взагалі*

5
□ *Відмовляюся відповідати*

999

Примітка: *Якщо відповідь **"Не вживав/ла взагалі"**, перейдіть, будь ласка, до **запитання 24.***

23. Чи доводилося Вам протягом 10 років до того моменту коли Ви дізналися, що у Вас ВІЛ, вживати ін'єкційним шляхом наступні наркотичні речовини?

23.1. ОПІАТИ (*Будь ласка, відмітьте все, що відповідає***)**

□ Дезоморфін ("крокодил", "електроширка")

1

□ Трамадол/трамал

2

□ Героїн

3

□ Екстракт опію в рідкому стані (“ширка”, “чорна”)

4

□ Вуличний метадон

5

□ Вуличний бупренорфін

6

□ Морфій

7

□ *Інші опіати, які саме*:_________________________?

8

□ *Не вживав/ла опіати*

9

23.2. СТИМУЛЯТОРИ (*Будь ласка, відмітьте все, що відповідає***)**

□
Кокаїн

1
□ Амфетамін ("фен") (у вигляді
порошку)

2
□ Метамфетамін у вигляді порошку
(кристалічний)

3
□ Метамфетамін у вигляді розчину ("вінт", "первінтин") - *препарати, які виготовляються з додаванням йоду та червоного фосфору з ліків, які містять ефедрин або*
псевдоефедрин

4
□ Меткатінон ("джефф") - *препарати, які виготовляються з додаванням марганцю з ліків, які містять*
ефедрин

5
□ Катінон ("бовтушка", "мулька") - *препарати, які виготовляються з додаванням марганцю з ліків на основі фенілпропаноламіну*
(PPA)

6
□ "Сіль для
ванн"

7
□ *Інші стимулятори, які*
саме:_________________________?

8

□ *Не вживав/ла стимулятори*

9

23.3. АПТЕЧНІ АБО ЗМІШАНІ (*Будь ласка, відмітьте все, що відповідає***)**

□ Аптечні наркотики (трапікамід, риназолін, каліпсол, кетамін) - *препарати, що купуються в аптеці та не потребують спеціального приготування для вживання з нетерапевтичною метою*

1

□ Аптечні седативні препарати («соннат» (зопіклон), димедрол)

2

□ Декілька речовин в одному шприці (опіати і барбітура - димедрол, транквілізатори та снодійні)

3

□ *Інші ін'єкційні наркотики, які саме*:____________________?

4

□ *Не вживав/ла*

5

24. Протягом 10 років до того моменту, коли Ви дізналися, що у Вас ВІЛ, чи вводили Вам (або чи вводили Ви собі самі) будь-які речовини нестерильним або вже бувшим у вжитку шприцом?

□ Так

1

□ Ні

2

□

Невідомо

998

□ *Відмовляюся відповідати*

999

25. Протягом 10 років до того моменту коли Ви дізналися, що у Вас ВІЛ, чи були у вашому житті випадки, коли….

25.1. ….Ви отримувати глибокі ушкодження шкіри голками або ножами (*наприклад, татуювання, нанесення рубців, релігійні обряди*)?

□
Так

1

□
Ні

2

□
Невідомо

998

□ *Відмовляюся відповідати*

999

25.2. ….Ви стикалися з попаданням крові іншої людини на Вашу ушкоджену шкіру та/або слизові оболонки?

□
Так

1

□
Ні

2

□
Невідомо

998

□ *Відмовляюся відповідати*

999

26. Наступні запитання про медичні процедури, які Ви проходили <u>протягом 10 років до</u>

<u>того моменту</u>, коли Ви дізналися, що у Вас ВІЛ.

26.1. Чи робили Вам переливання крові або продуктів крові, таких як фактори згортання крові? Наприклад, у зв'язку з гемофілією чи іншими порушеннями згортання крові.

- *Фактори згортання крові - речовини у плазмі крові і тромбоцитах, які забезпечують згортання крові.*

□ Так 1

□ Ні 2

□ *Невідомо* 998

□ *Відмовляюся відповідати* 999

Якщо ТАК, то в якому році / роках? ____

□ *Не пам'ятаю рік/роки*..................................999

26.2. Чи робили Вам операції з пересадки/трансплантації органів? *Наприклад, нирка, серце або легені, рогівка або кістковий трансплантат.*

□ Так 1

□ Ні 2

□ *Невідомо* 998

□ *Відмовляюся відповідати* 999

Якщо ТАК, то в якому році / роках? ____

□ *Не пам'ятаю рік/роки*..................................999

26.3. ПРИМІТКА: Запитання тільки для жінок!

Чи проходили Ви процедуру штучного запліднення, намагаючись завагітніти?

□ Так 1
□ Ні 2
□ *Невідомо* 998
□ *Відмовляюся відповідати* 999

Якщо ТАК, то в якому році / роках? ____

□ *Не пам'ятаю рік/роки*................................999

27. До того моменту, як Ви дізналися, що у Вас ВІЛ-інфекція, чи хворіли Ви на такі захворювання?

Будь ласка, відмітьте все, що відповідає, та роки, коли хворіли. Запишіть 999 у полі "Рік / роки", якщо не пам'ятаєте, коли це було.		**Рік / роки, коли хворіли**
□ Гепатит В..	1	____________
□ Гепатит С..	2	____________
□ Гепатит, який саме - невідомо..	3	____________
□ Гонорея (у тому числі ротової порожнини або горла)	4	____________
□ Ректальна гонорея (гонорея прямої кишки)	5	____________
□ Сифіліс..	6	____________
□ Генітальний (статевий) герпес	7	____________
□ Проктит (запалення слизової оболонки прямої кишки)	8	____________
□ Будь-яке інше захворювання, що передається статевим шляхом..	9	____________
□ *Не хворів/ла на такі захворювання*...	10	
□ *Відмовляюся відповідати*...	999	

28. Чи вакцинувались Ви від гепатиту В за останні 5 років?

□
Так

1
□
Ні

2
□
Невідомо

998
□ *Відмовляюся відповідати*

999

29. **Чи працювали/працюєте Ви у закладах охорони здоров'я чи клінічних лабораторіях?**

□
Так

1
□
Ні

2
□ *Відмовляюся відповідати*

999

30. **Чи перебували Ви коли-небудь в місцях позбавлення волі (в слідчому ізоляторі (СІЗО), виправній колонії чи камері попереднього ув'язнення (КПУ))?**

□
Так

1
□
Ні

2
□ *Відмовляюся відповідати*

999

31. **Коли, на Вашу думку, Ви могли інфікуватись ВІЛ?**

Якщо менше року, скільки МІСЯЦІВ тому це сталося?

Якщо більше 12 місяців, скільки РОКІВ тому це сталося?

32. **Як, на Вашу думку, Ви інфікувалися ВІЛ?**

□ Статевим шляхом, при *гетеросексуальних* контактах з ВІЛ-інфікованим *партнером, який НЕ вживав наркотики*
ін'єкційно

1
□ Статевим шляхом, при *гомосексуальних* контактах з ВІЛ-інфікованим *партнером, який НЕ вживав наркотики*
ін'єкційно

2
□ Статевим шляхом, при *гетеросексуальних* контактах з ВІЛ-інфікованим *партнером, що вживав наркотики*
ін'єкційно

3
□ Статевим шляхом, при *гомосексуальних* контактах з ВІЛ-інфікованим *партнером, що вживав наркотики*
ін'єкційно

4
□ Через використання спільних шприців / голок під час прийому наркотиків ін'єкційним
шляхом

5
□ Через використання нестерильних медичних інструментів у лікарні або поліклініці

6

□ Під час переливання (Вам) крові, що була інфікована ВІЛ

7

□ Під час пересадки (Вам) органів / тканин від ВІЛ-інфікованого донора

8

□ Через використання нестерильних інструментів, коли робили татуювання або пірсинг

9

□ У результаті аварійної ситуації на робочому місці (під час роботи в лабораторії, хірургічних втручань, забору крові, медичних маніпуляцій тощо)

10

□ *Інше, що саме*:____?

11

КОМЕНТАР 5: Дякуємо за час, що Ви знайшли для участі у даному дослідженні. Інформація дуже допоможе нам дізнатися більше про ВІЛ-інфекцію та шляхи інфікування ВІЛ в Україні.

		**:**		
(ГГ:ХХ)				

Час завершення інтерв'ю:
